# TetR-like regulator BP1026B_II1561 controls aromatic amino acid biosynthesis and intracellular pathogenesis in *Burkholderia pseudomallei*

**DOI:** 10.3389/fmicb.2024.1441330

**Published:** 2024-08-15

**Authors:** Ian A. McMillan, Michael H. Norris, Yun Heacock-Kang, Jan Zarzycki-Siek, Zhenxin Sun, Brooke A. Hartney, Liliana K. Filipowska, M. Nurul Islam, Dean C. Crick, Bradley R. Borlee, Tung T. Hoang

**Affiliations:** ^1^School of Life Sciences, University of Hawaiʻi at Mānoa, Honolulu, HI, United States; ^2^Pathogen Analysis and Translational Health Group, School of Life Sciences, University of Hawaiʻi at Mānoa, Honolulu, HI, United States; ^3^Department of Microbiology, Immunology, and Pathology, Colorado State University, Fort Collins, CO, United States; ^4^Department of Chemistry, Biochemistry, and Physics, South Dakota State University, Brookings, SD, United States

**Keywords:** bacteriology, *Burkholderia pseudomallei*, melioidosis, transcriptional regulation, pathogenesis

## Abstract

*Burkholderia pseudomallei* (*Bp*) causes the tropical disease melioidosis that afflicts an estimated 165,000 people each year. *Bp* is a facultative intracellular pathogen that transits through distinct intracellular stages including attachment to host cells, invasion through the endocytic pathway, escape from the endosome, replication in the cytoplasm, generation of protrusions towards neighboring cells, and host cell fusion allowing *Bp* infection to spread without exiting the intracellular environment. We have identified a TetR-like transcriptional regulator, BP1026B_II1561, that is up-regulated during the late stages of infection as *Bp* protrudes toward neighboring cells. We have characterized BP1026B_II1561 and determined that it has a role in pathogenesis. A deletional mutant of *BP1026B_II1561* is attenuated in RAW264.7 macrophage and BALB/c mouse models of infection. Using RNA-seq, we found that BP1026B_II1561 controls secondary metabolite biosynthesis, fatty acid degradation, and propanoate metabolism. In addition, we identified that BP1026B_II1561 directly controls expression of an outer membrane porin and genes in the shikimate biosynthetic pathway using ChIP-seq. Transposon mutants of genes within the BP1026B_II1561 regulon show defects during intracellular replication in RAW264.7 cells confirming the role of this transcriptional regulator and the pathways it controls in pathogenesis. BP1026B_II1561 also up-regulates the majority of the enzymes in shikimate and tryptophan biosynthetic pathways, suggesting their importance for *Bp* physiology. To investigate this, we tested fluorinated analogs of anthranilate and tryptophan, intermediates and products of the shikimate and tryptophan biosynthetic pathways, respectively, and showed inhibition of *Bp* growth at nanomolar concentrations. The expression of these pathways by BP1026b_II1561 and during intracellular infection combined with the inhibition of *Bp* growth by fluorotryptophan/anthranilate highlights these pathways as potential targets for therapeutic intervention against melioidosis. In the present study, we have identified BP1026B_II1561 as a critical transcriptional regulator for *Bp* pathogenesis and partially characterized its role during host cell infection.

## Introduction

*Burkholderia pseudomallei* (*Bp*) is a Tier 1 select agent that causes the tropical disease melioidosis (Wiersinga et al., 2018). Melioidosis is predicted to cause ~89,000 deaths annually through a wide array of clinical manifestations that are often misdiagnosed (Hoffmaster et al., 2015; Limmathurotsakul et al., 2016). Acute, chronic, and sometimes latent forms of melioidosis are often found in patients of endemic areas with the majority of acute cases presenting with life threatening sepsis (Currie et al., 2010). Cases of melioidosis in the United States have been linked to imported consumer products and, more recently, domestic environmental exposures expanding the endemic region of *Bp* to include the Southeastern USA (Dawson et al., 2021; Environmental Samples, 2022; Gee et al., 2022). Pneumonia and localized abscesses are common presentations although many other nonspecific clinical signs can confound diagnosis making laboratory tests critical (Hoffmaster et al., 2015). Treatment is broken down into an initial intensive phase that consists of intravenous ceftazidime, meropenem, or imipenem followed by an eradication phase consisting of either trimethoprim-sulfamethoxazole, amoxicillin clavulanate, or doxycycline (Wiersinga et al., 2012). Although modern antimicrobial treatment reduced mortality rates by half (White et al., 1989), *Bp* is intrinsically resistant to antimicrobials through numerous mechanisms and resistance can develop during treatment (Chantratita et al., 2011; Sarovich et al., 2012; Rhodes and Schweizer, 2016). Due to this and the lack of an approved vaccine, the development of novel therapeutic treatments is critical to reducing the global burden of melioidosis.

Melioidosis is acquired through inhalation, ingestion, or inoculation from environments containing *Bp* (Wiersinga et al., 2012). *Bp* is a facultative intracellular pathogen that lives freely within the environment and is associated with the rhizosphere (Kaestli et al., 2012). Infection is thought of as opportunistic in immunocompetent humans (Currie et al., 2010) and intracellular virulence mechanisms could have evolved through associations with environmental eukaryotes like *Acanthamoeba* species (Inglis et al., 2000). *Bp* can infect many cells and tissue types throughout the human body including, but not limited to, cells of the lungs, liver, spleen, skin, bone/joint, gastrointestinal organs, and central nervous system (Wiersinga et al., 2018). *Bp* requires many virulence factors to infect this variety of cell types. Described and characterized virulence factors include lipopolysaccharide (Tuanyok et al., 2012; Norris et al., 2017, 2018), capsule polysaccharide (Reckseidler-Zenteno et al., 2010; Woodman et al., 2012; Mongkolrob et al., 2015), type III secretion systems (T3SS) (Stevens et al., 2002; Warawa and Woods, 2005; Lee et al., 2010; French et al., 2011; Gong et al., 2011), type VI secretion systems (T6SS) (Shalom et al., 2007; Burtnick et al., 2011; Schwarz et al., 2014; Toesca et al., 2014; Lim et al., 2015), host cell actin polymerization through BimA (Stevens et al., 2005; Benanti et al., 2015), and numerous others (Wiersinga et al., 2018). The roles of these virulence factors have been assigned to specific spatial and temporal locations during infection. Briefly, *Bp* attaches to host cells, is internalized, and uses the *Burkholderia* secretion apparatus (T3SS_Bsa_) to escape the vacuole to gain entry into the cytoplasm (Gong et al., 2011). Within the cytoplasm, *Bp* can replicate and move freely by polymerizing host cell actin with a type 5 autotransporter BimA through molecular mimickry (Benanti et al., 2015) or, in some cases, using its lateral flagella (French et al., 2011). When bacterial cell density increases and/or nutrients become limited, *Bp* fuses host cells using a T6SS to generate multinucleated giant cells (MNGCs) (French et al., 2011; Toesca et al., 2014; Lim et al., 2015). Recently, our group identified thousands of genes that are differentially regulated through the *Bp* intracellular lifecycle (Heacock-Kang et al., 2021), suggesting that a sophisticated regulatory network exists to coordinate pathogenesis. In addition, the identification of Intracellular Pathogenesis Regulator A (IprA), a PadR family transcription factor that is differentially expressed during host cell intracellular infection and required for complete pathogenesis in cell culture and BALB/c mice, is further evidence indicating that a complex intracellular regulatory network exsists (McMillan et al., 2021). Here, we present data to understand the role of a transcriptional regulator, BP1026B_II1561, and how it is involved in *Bp* pathogenesis and intracellular survival.

## Results

### BP1026B_II1561 is up-regulated as *Bp* protrudes toward neighboring cells

**Figure 1:**
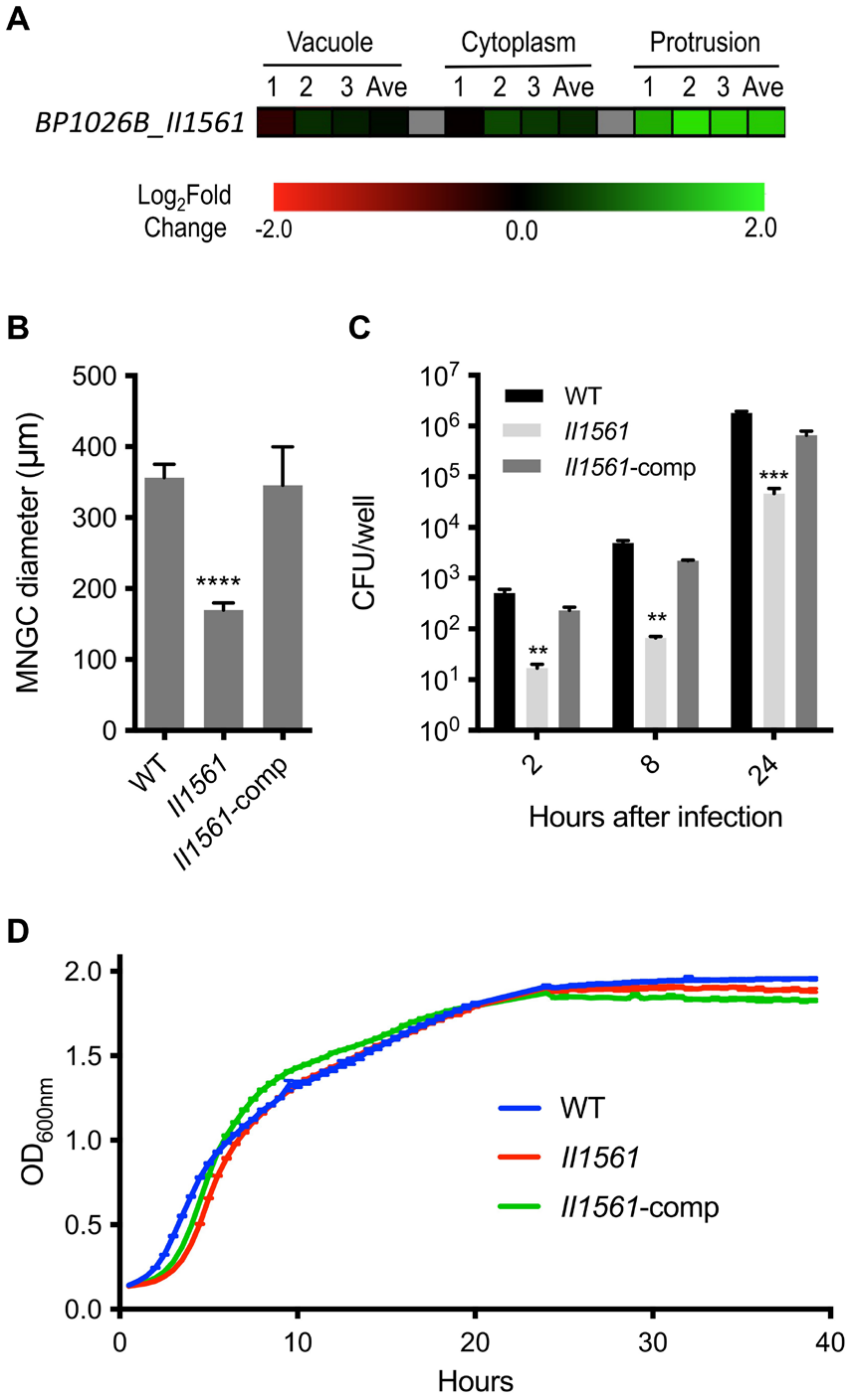
Differential regulation of BP1026B_II1561 during cell infection leads to defects in pathogenesis. **(A)** BP1026B_II1561 is up-regulated while *Bp* is protruding towards neighboring cells. Expression data represent three biological replicates (1, 2, and 3) and the average (Ave) with a green-black-red color scale used to represent up- and down-regulation based on log_2_FC. **(B)** An in-frame deletion mutant of BP1026B_II1561 (II1561) was unable to form MNGCs of similar diameter to wild type *Bp* 1026b (WT) in RAW264.7 cells. Complementation of the mutant (II1561-comp) recovered this defect. **(C)** The BP1026B_II1561 mutant was significantly impaired during intracellular replication at 2, 8, and 24 h post infection in RAW264.7 cells, and the complement was able to recover the defect. **(D)** The BP1026B_II1561 mutant and its complement were able to grow similarly to wild type in LB broth. Data in bar graphs and growth curves represent means ± s.e.m. and are analyzed via unpaired *t*-test comparing to the WT group. *p* values presented are as follows: ** <0.01, *** < 0.001, **** < 0.0001.

*Bp* differentially regulates 1,953 bacterial genes during intracellular infection of RAW264.7 murine macrophages (Heacock-Kang et al., 2021). The number of genes operating within each intracellular niche and the coordination between spatial locations indicates that a sophisticated mechanism for gene regulation must exist. A PadR-type regulator, IprA, was recently identified to be differentially expressed during intracellular infection and tied to *Bp* pathogenesis (McMillan et al., 2021). Many other putative transcriptional regulators are encoded within the *Bp* genome including BP1026B_II1561. BP1026B_II1561 is predicted to be part of a transcriptional regulator family with 654 orthologous group members within the *Burkholderia* genus, suggesting a conserved role (Winsor et al., 2008). Structurally, BP1026B_II1561 has a low complexity/disorder region on the N-terminus (AA 1–36), an N-terminal TetR domain (Pfam: PF00440, AA40-86), and a C-terminal TetR domain (Pfam: PF17938, AA108-226) (Sonnhammer et al., 1997; Winsor et al., 2008). During intracellular infection, BP1026B_II1561 is highly expressed in the later stages of cellular infection as *Bp* protrudes towards neighboring cells (Figure 1A) (Heacock-Kang et al., 2021). The major function of BP1026B_II1561 as a potential transcriptional regulator is likely isolated to the membrane protrusion compartment. However, it may have potential implications during other stages of intracellular infection, prompting us to investigate the role it plays during RAW264.7 murine macrophage pathogenesis.

### A BP1026B_II1561 mutant is defective in multinucleated giant cell (MNGC) formation, intracellular replication, and *in vivo* pathogenesis

Because transcriptional regulators play an important role in many cellular processes and BP1026B_II1561 is differentially expressed during host cell transit, we hypothesized that BP1026B_II1561 will be critical for intracellular infection. To answer this, we generated a deletion mutant of BP1026B_II1561 to test its role during pathogenesis. When infected with the *BP1026B_II1561* mutant, RAW264.7 cells form smaller diameter MNGCs when compared to cells infected with wild type *Bp* 1026b (Figure 1B). This defect was complemented when *BP1026B_II1561* was expressed *in trans* in the mutant strain. To further characterize the role of BP1026B_II1561 during RAW264.7 cell infection, the number of intracellular bacteria was determined at various time points. At 2, 8, and 24 h post-infection, the *BP1026B_II1561* mutant showed a significant decrease in intracellular replication when compared to wild type *Bp* 1026b (Figure 1C). When grown in LB broth, the *BP1026B_II1561* mutant and its complement showed comparable growth kinetics compared to wild type *Bp* 1026b, indicating that the defects during intracellular infection can be tied to pathogenesis rather than *in vitro* fitness (Figure 1D). These data indicate that BP1026B_II1561 is important for intracellular replication and cell–cell spread during RAW264.7 cell infection.

**Figure 2:**
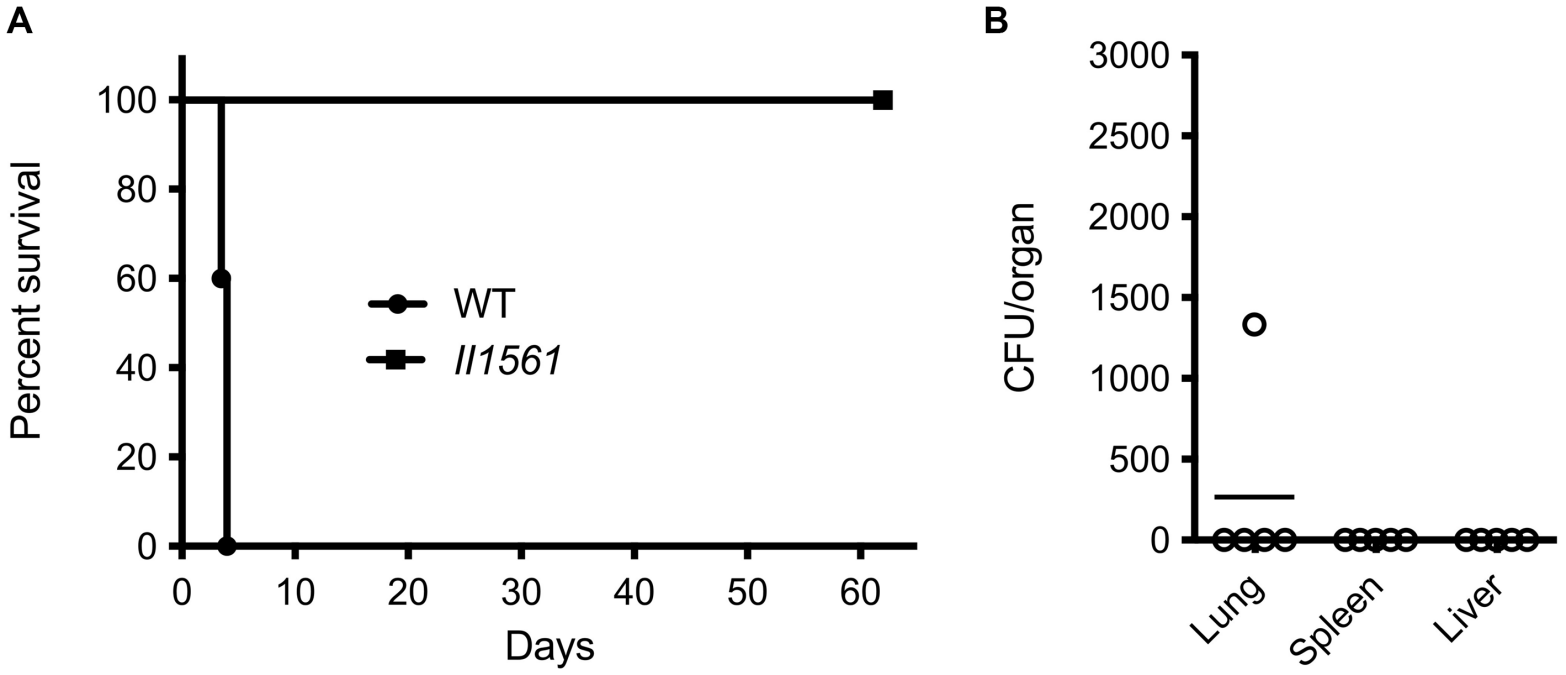
A *BP1026B_II1561* mutant is highly attenuated in the BALB/c mouse infection model. **(A)** BALB/c mice were infected with 4,500 CFU of wild type *Bp* 1026b (WT) or the *BP1026B_II1561* mutant (*II1561*). Mice infected with the *BP1026B_II1561* mutant were able to survive for the duration of the study, while mice infected with wild type *Bp* 1026b showed 100% mortality by day five. Survival of mice infected with the *BP1026B_II1561* mutant was significant, *p* = 0.0031 via log-rank (Mantel-Cox) test, when compared to the survival of mice infected with wild type *Bp* 1026b. **(B)** Bacterial burdens from the lungs, liver, and spleen of surviving mice were determined to show significant clearance of the mutant strain with only one mouse having persistent infection in the lungs.

To determine the role of BP1026B_II1561 in a model of murine melioidosis, we utilized the highly susceptible BALB/c mouse model to test if the *BP1026B_II1561* mutant showed reduced levels of mortality. BABL/c mice were infected via the intranasal route with 4,500 colony-forming units (CFU) of either wild type *Bp* 1026b or the *BP1026B_II1561* mutant. While all mice infected with wild type *Bp* 1026b (*n* = 5) showed significant signs of infection and 100% mortality by day four, all mice infected with the *BP1026B_II1561* mutant (*n* = 5) were able to survive for 62 days when the study was terminated (Figure 2A). Upon study termination all mice infected with the *BP1026B_II1561* mutant were euthanized and lungs, livers, and spleens were harvested for bacterial burden analysis. Four mice appeared to have cleared the *BP1026B_II1561* mutant and one mouse had a persistent infection within the lungs (Figure 2B). Collectively, BP1026B_II1561 is important for pathogenesis in RAW264.7 cells and BALB/c mice. To better understand why this transcriptional regulator is critical for pathogenesis, we sought to discover the regulation network of BP1026B_II1561 through RNA-seq analysis.

### Hypothetical proteins and secondary metabolites are controlled by BP1026B_II1561

**Figure 3:**
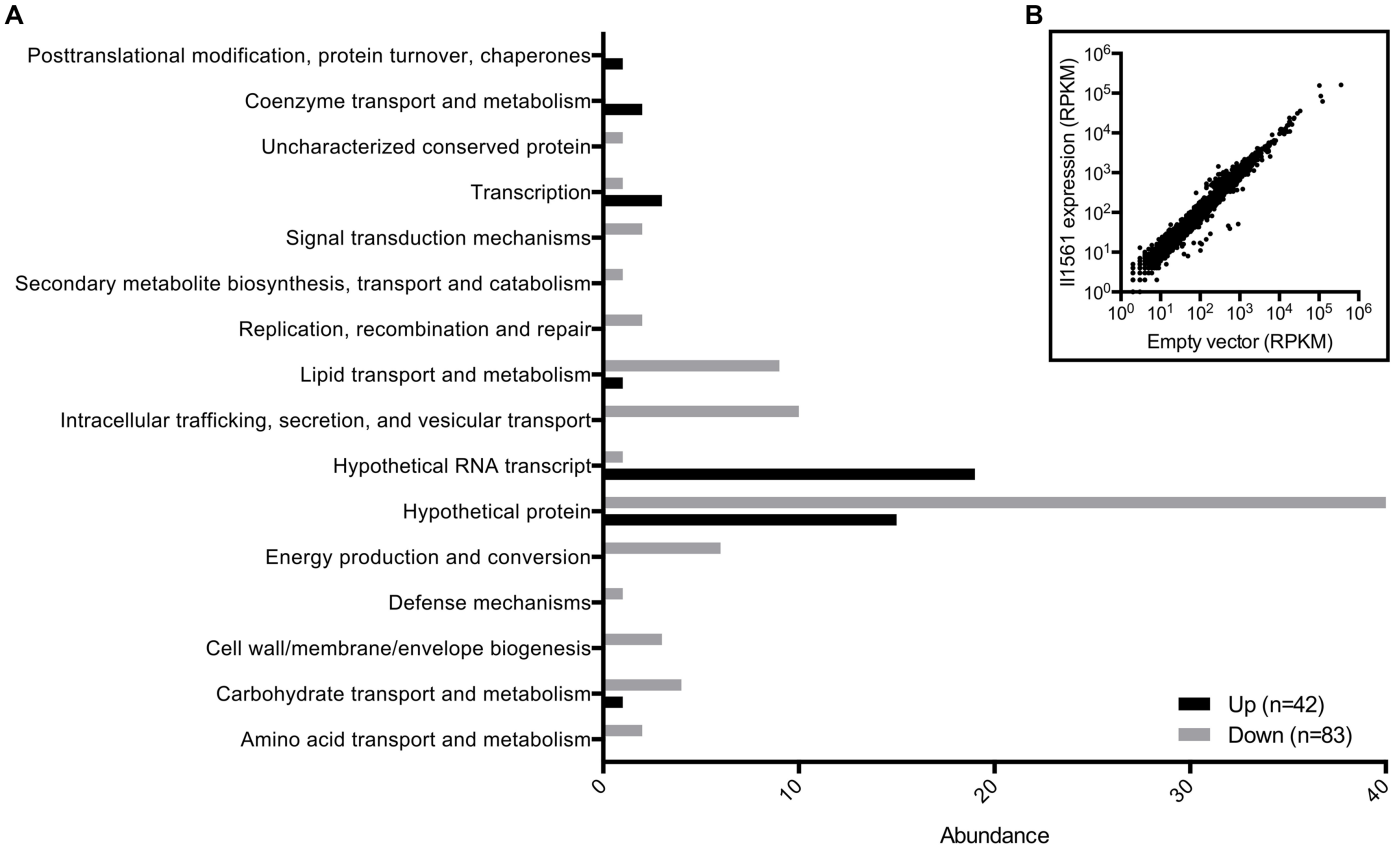
BP1026B_II1561 down-regulates 83 genes and up-regulates 42 genes. **(A)** COG functional predictions of genes with a q-value of <0.01 and a log_2_FC of ≥1 or ≤ −1 show the general landscape of the BP1026B_II1561 regulation network. A large proportion of genes have no known function. **(B)** Scatter plot of all *Bp* genes comparing expression of BP1026B_II1561 and the empty vector control.

To further investigate the function of this transcriptional regulator, we determined how the expression of BP1026B_II1561 changes the transcriptome of *Bp* 1026b. This identified the potential pathways/genes controlled by BP1026B_II1561 to further characterize its role during pathogenesis. BP1026B_II1561 was expressed, via IPTG inducible pAM3GIQ-3xTY1-*BP1026B_II1561*, under the same conditions that were used to complement the mutation during infection, followed by total RNA extraction and Illumina sequencing (McMillan et al., 2021). The data was analyzed by Rockhopper (McClure et al., 2013) identifying the BP1026B_II1561 regulatory network (Figure 3B, Supplementary Table S1). We selected genes with a *q*-value <0.01 and a log_2_ fold-change (log_2_FC) of ≥1 or ≤ −1 to analyze further (Figure 3A). In total, 42 genes are up-regulated by BP1026B_II1561 while 83 genes are down-regulated (Figure 3A). When we analyzed these genes by their COG functional groups, 76 are characterized as hypothetical proteins, hypothetical RNA transcripts, or uncharacterized conserved proteins, highlighting the unknown nature of the pathways/genes controlled by BP1026B_II1561 (Figure 3A). Of these, 42 are down-regulated and 34 are up-regulated by BP1026B_II1561. Many other general pathways are down regulated by BP1026B_II1561 including lipid, carbohydrate, amino acid metabolism and transport, energy production and conversion, and cell wall/membrane/envelope biogenesis (Figure 3A). In addition, it appears that mechanisms of virulence are also down-regulated including COG functional categories of intracellular trafficking, secretion, and vesicular transport, secondary metabolite biosynthesis, transport, and catabolism, and defense mechanisms (Figure 3A). Although COG functional predictions can give us a general idea of what known pathways are being controlled by BP1026B_II1561, they are limited because much of the *Bp* genome has not been characterized.

For another perspective on what genes/pathways are being controlled by BP1026B_II1561, we analyzed the entire RNA-seq data set using WoPPER (Puccio et al., 2017). WoPPER analysis allowed us to visualize regions of DNA controlled by BP1026B_II1561 by comparing localized strand-separated expression across the entire data set. While our COG analysis focused on single genes that fit our criteria (*q*-value <0.01 and log_2_FC of ≥1 or ≤ −1), WoPPER analysis does not, and will include regions of genes with minor changes in expression. BP1026B_II1561 controls 40 gene clusters on chromosome I (Supplementary Figure S1) and 50 gene clusters on chromosome II (Supplementary Figure S2). In contrast to the COG analysis, WoPPER identified 53 clusters of genes that are up-regulated by BP1026B_II1561 while 37 clusters of genes are down-regulated (Supplementary Figures S1, S2). Clusters of known secondary metabolites are controlled by BP1026B_II1561 including malleobactin (Alice et al., 2006), malleilactone (Biggins et al., 2012), pyochelin (Biggins et al., 2014), and malleipeptin (Biggins et al., 2014). Gene cluster 19 on chromosome I is up-regulated by BP1026B_II1561 and includes the non-ribosomal peptide synthase (NRPS) malleobactin, a siderophore (Supplementary Figure S1). Another siderophore, pyochelin, is located in the up-regulated gene cluster 15 on chromosome II (Supplementary Figure S2). While these gene clusters are important for iron acquisition, they are also unnecessary for murine pathogenesis (Kvitko et al., 2012). Malleilactone on the other hand is a siderophore that has been tied to pathogenesis in *Caenorhabditis elegans* and *Dictyostelium discoideum* (Biggins et al., 2012; Klaus et al., 2018). Malleilactone is up-regulated by BP1026B_II1561 in gene cluster 6 on chromosome II (Supplementary Figure S2). BP1026B_II1561 appears to control genes and pathways that are critical for iron-limiting environments. Other secondary metabolite gene clusters are also controlled by BP1026B_II1561. Malleipeptin is down regulated in cluster 37 on chromosome II by BP1026B_II1561 (Supplementary Figure S2). Malleipeptin is a lipopeptide encoded by five genes (*BP1026B_II1742-II1746*) and is required for pathogenesis in mice (Biggins et al., 2014). While malleipeptin has been linked to bacterial invasion (Biggins et al., 2014), no role during later stages of infection has been identified. Our data fit into this model because we see up-regulation of BP1026B_II1561 during the later stages of infection (Figure 1A), leading to repression of malleipeptin. In addition to these secondary metabolites, a polyketide synthase/peptide synthetase cluster of genes around *BP1026B_II1265* are down-regulated by BP1026B_II1561 (cluster 27, Supplementary Figure S2). This cluster of genes has yet to be characterized but could play a role during *Bp* intracellular survival (Biggins et al., 2014). The variation in expression of *Bp* secondary metabolites suggests that these compounds could be critical for intracellular infection. The combination of both COG and WoPPER analysis has helped us define a general regulatory role of BP1026B_II1561 within the *Bp* 1026b genome.

### BP1026B_II1561 controls genes that have potential roles during intracellular pathogenesis

**Figure 4:**
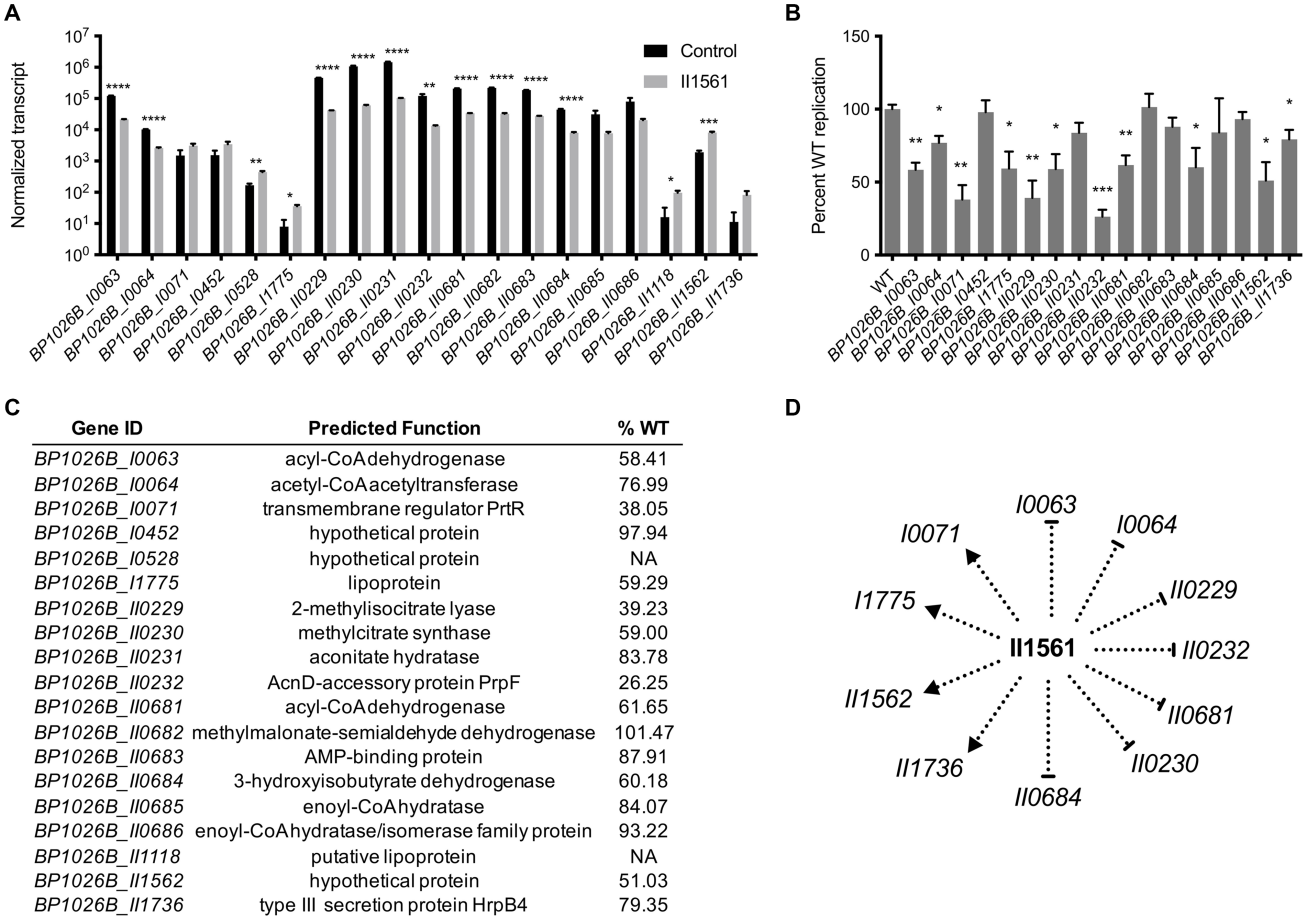
General metabolic pathways controlled by BP1026B_II1561 contribute to pathogenesis in RAW264.7 cells. **(A)** BP1026B_II1561 up-regulates seven genes with a log_2_FC of ≥2 and down-regulates 12 genes with a log_2_FC of ≤ −2. Black bars are normalized transcript levels of the empty vector control and grey bars are normalized transcript levels in the presence of BP1026B_II1561. **(B)** Transposon mutants of indicated genes show various levels of intracellular replication in RAW264.7 cells at 24 h post infection when compared to wild type *Bp* 1026b (WT). **(C)** Predicted functions of genes controlled by BP1026B_II1561. Average percent wild type replication (%WT) of transposon mutants identifies genes important for intracellular survival. **(D)** Model of the genes controlled by BP1026B_II1561 that contribute to intracellular survival in RAW264.7 cells. Pointed arrows represent activation and flat arrows represent repression. Data in bar graphs represent means ± s.e.m. and are analyzed via unpaired *t*-test. *p* values presented are as follows: * < 0.05, ** < 0.01, *** < 0.001, **** < 0.0001.

To better characterize the pathogenic role that BP1026B_II1561 plays during intracellular infection, genes with a log_2_FC of ≥2 or ≤ −2 were targeted for mutant analysis (Supplementary Table S1). Twelve of these genes are down-regulated and seven are up-regulated by BP1026B_II1561 (Figure 4A). These mutants were obtained from the sequence-defined transposon mutant library of *Bp* 1026b (Borlee et al., 2017) and the T24 transposon insertion was confirmed by sequencing. No transposon mutants with insertions in genes *BP1026B_I0528* or *BP1026B_II1118* were present. To characterize the pathogenic role these genes have during infection, RAW264.7 murine macrophages were infected with the transposon mutants and wild type *Bp* 1026b followed by determination of the intracellular bacterial burden. Transposon mutants tested were able to replicate at 26.25–101.47% of wild type over the course of the infection (Figures 4B,C). Some mutants, *BP1026B_I0452*, *BP1026B_II0231*, *BP1026B_II0682*, *BP1026B_II0683*, *BP1026B_II0685*, and *BP1026B_II0686*, showed similar intracellular replication to wild type *Bp* 1026b indicating less essential roles during infection (Figure 4C). Interestingly, genes involved in fatty acid metabolism, *BP1026B_I0063* and *BP1026B_I0064*, show 58.41 and 76.99% wild type replication, respectively (Figure 4C). These two genes encode acyl-CoA dehydrogenase and acetyl-CoA acetyltransferase and are down-regulated by BP1026B_II1561 with log_2_FCs of-2.5 and-2 (Figure 4A, Supplementary Table S1). In addition to this, *BP1026B_I0065*, the gene encoding the alpha subunit of the fatty acid oxidation complex, is also down-regulated by BP1026B_II1561 (log2FC-1.5, Supplementary Table S1). These data indicate that BP1026B_II1561 down-regulates fatty acid metabolism and that some of the enzymes in this pathway are important for intracellular survival.

*BP1026B_I0071* encodes a transmembrane regulator protein, PrtR, that is up-regulated by BP1026B_II1561 with a log_2_FC of 2 (Figure 4A, Supplementary Table S1). PrtR sits downstream from an extracytoplasmic function sigma factor (ECF), *BP1026B_I0072*/*prtI*, with no known function. While *BP1026B_I0071* is up-regulated, *BP1026B_I0072* is only slightly up-regulated by BP1026B_II1561 with a log_2_FC of 0.48 (Supplementary Table S1). A transposon mutant in *BP1026B_I0071* reduced intracellular replication to 38% of wild type *Bp* 1026b, suggesting a role during intracellular replication and pathogenesis (Figures 4B,C). *BP1026B_I1775* encodes a potential lipoprotein that is up-regulated by BP1026B_II1561 and a mutant of this gene replicates at 59.29% of wild type in RAW264.7 cells (Figures 4A–C). The function of this lipoprotein is unknown, but our results suggest that it is important for pathogenesis. Genes involved in propanoate metabolism are also controlled by BP1026B_II1561. *BP1026B_II0229-II0232* are highly down-regulated with log_2_FCs between −3.2 to −4.2 (Figure 4A, Supplementary Table S1). These genes catabolize propanoyl-CoA to succinate and pyruvate that can feed into the citric acid cycle (Kanehisa and Goto, 2000). Mutants of *BP1026B_II0229*, *BP1026B_II0230*, and *BP1026B_II0232* showed significant decreases during intracellular replication (Figure 4B). Strikingly, the transposon mutant of *BP1026B_II0232* was reduced to replicate at 26.25% of wild type *Bp* 1026b in RAW26.7 macrophages (Figures 4B,C).

Other genes controlled by BP1026B_II1561 have known functions in general metabolic pathways and are linked to *BP1026B_II0229-II0232*. *BP1026B_II0681-II0686* are down-regulated by BP1026b_II1561 ranging from a log_2_FC of-2.02 to-2.77 (Figure 4A, Supplementary Table S1). *BP1026B_II0682* encodes methylmalonate-semialdehyde dehydrogenase and catalyzes the reaction of either methymalonate semialdehyde to propanoyl-CoA or malonate semialdehyde to acetyl-CoA (Kanehisa and Goto, 2000). A transposon mutant in this gene replicates in RAW264.7 cells at 101.5% of wild type indicating that its loss can be compensated for during intracellular infection (Figures 4B,C). BP1026B_II0683 is an AMP-binding protein that is predicted to catalyze the reactions of acetate to acetyl-CoA or propanoate to propanoyl-CoA (Kanehisa and Goto, 2000), and the mutant of this gene replicates at 87.91% of wild type (Figures 4B,C). *BP1026B_II0684* encodes 3-hydroxyisobutyrate dehydrogenase that catalyzes the reaction of hydroxyl isobutyrate to methylmalonate semialdehyde that can then be converted to propanoyl-CoA by *BP1026B_II0682*. Propanoyl-CoA is further degraded by BP1026B_II0229-II0232 to succinate and pyruvate (Kanehisa and Goto, 2000). Interestingly, *BP1026B_II0684* is important for intracellular survival because its mutant replicates at 60.18% of wild type in RAW264.7 cells (Figures 4B,C). Three genes, *BP1026B_II0681*, *BP1026B_II0685*, and *BP1026B_II0686*, that are involved in beta-oxidation during fatty acid metabolism are also down regulated by BP1026B_II1561 (Figure 4A). Of these genes, *BP1026B_II0685-II0686* mutants show no major defect when tested for intracellular replication while a mutant of *BP1026_II0681* replicated at 61.65% of wild type *Bp* (Figures 4B,C). *BP1026B_II1562* is a hypothetical gene that is up-regulated by BP1026B_II1561 with a log_2_FC of 2.11 (Figure 4A, Supplementary Table S1). While a mutant in *BP1026B_II1562* replicates at 51% of wild type (Figures 4B,C), it is only found in *Bp* strain 1026b and contains regions of disorder throughout its primary sequence (Winsor et al., 2008). *BP1026B_II1736* encodes a type III secretion protein HrpB4 that is up-regulated by BP1026B_II1561 (Figure 4A) and its mutant is impaired in intracellular replication (Figures 4B,C). Transposon mutants defective in RAW264.7 cell infection were grown *in vitro* and showed similar growth kinetics to wild type *Bp* indicating that the defects during intracellular replication were not due to decreased fitness (Supplementary Figure S3A). Through this analysis, we have identified general metabolic pathways and uncharacterized genes in the BP1026B_II1561 regulon that contribute to intracellular pathogenesis of RAW264.7 macrophages (Figure 4D).

### BP1026B_II1561 binds to two regions on chromosome II

**Figure 5:**
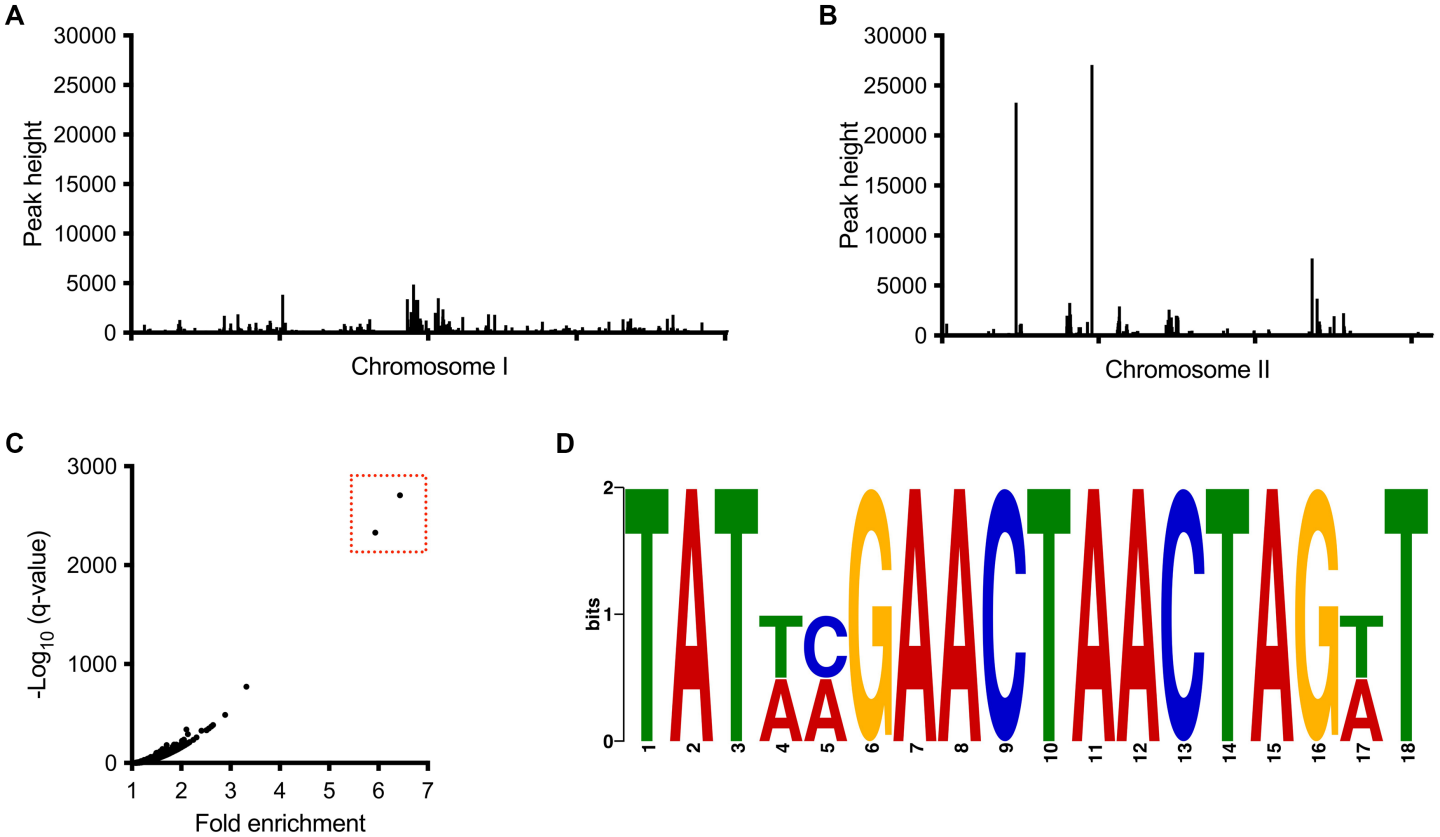
BP1026B_II1561 binds two regions of DNA on chromosome II. All peaks identified from the ChIP-seq experiment on chromosome I **(A)** and chromosome II **(B)** show that BP1026B_II1561 binds two regions on chromosome II. **(C)** Comparison of the –log_10_ of the *q*-value compared to fold enrichment further isolates two peaks (boxed in red) from the background. **(D)** Consensus region of DNA from the two peaks identified in ChIP-seq data found using MEME (Bailey and Elkan, 1994). The consensus binding region shows 15 out of 18 base pairs are shared between the two peaks.

To get a better understanding of the direct role of BP1026B_II1561 on transcription, we used ChIP-seq to define the direct binding sequences within the genome as previously described (Heacock-Kang et al., 2018a,b,c; McMillan et al., 2021). Immunoprecipitated DNA from a *Bp*82 strain expressing BP1026B_II1561 was compared to an empty vector control, which identified many peaks across both chromosomes when mapped to the *Bp* 1026b genome (Figures 5A,B). Two large peaks were identified with 5.9 and 6.4 fold enrichment on chromosome II (Figure 5B). Comparison of the fold enrichment and the false discovery rate (−log_10_ of the q-value) of both peaks further differentiates them from the background (Figure 5C). While BP1026B_II1561 had other binding regions located on chromosome II and several on chromosome I, the low fold enrichment and false discovery rate (−log_10_ of the q-value) indicates that these peaks are not major binding regions of BP1026B_II1561 under the conditions tested (Figures 5A–C). The BP1026B_II1561 binding peaks on chromosome II are 610 bp and 762 bp in length and these sequences were used to identify an 18 bp consensus binding sequence, TAT XXG AAC TAA CTA GXT, with 15 bp having 100% homology between both peaks using MEME (Bailey and Elkan, 1994) (Figure 5D). We believe that this is the region in which BP1026B_II1561 directly binds to activate/repress transcription.

**Figure 6:**
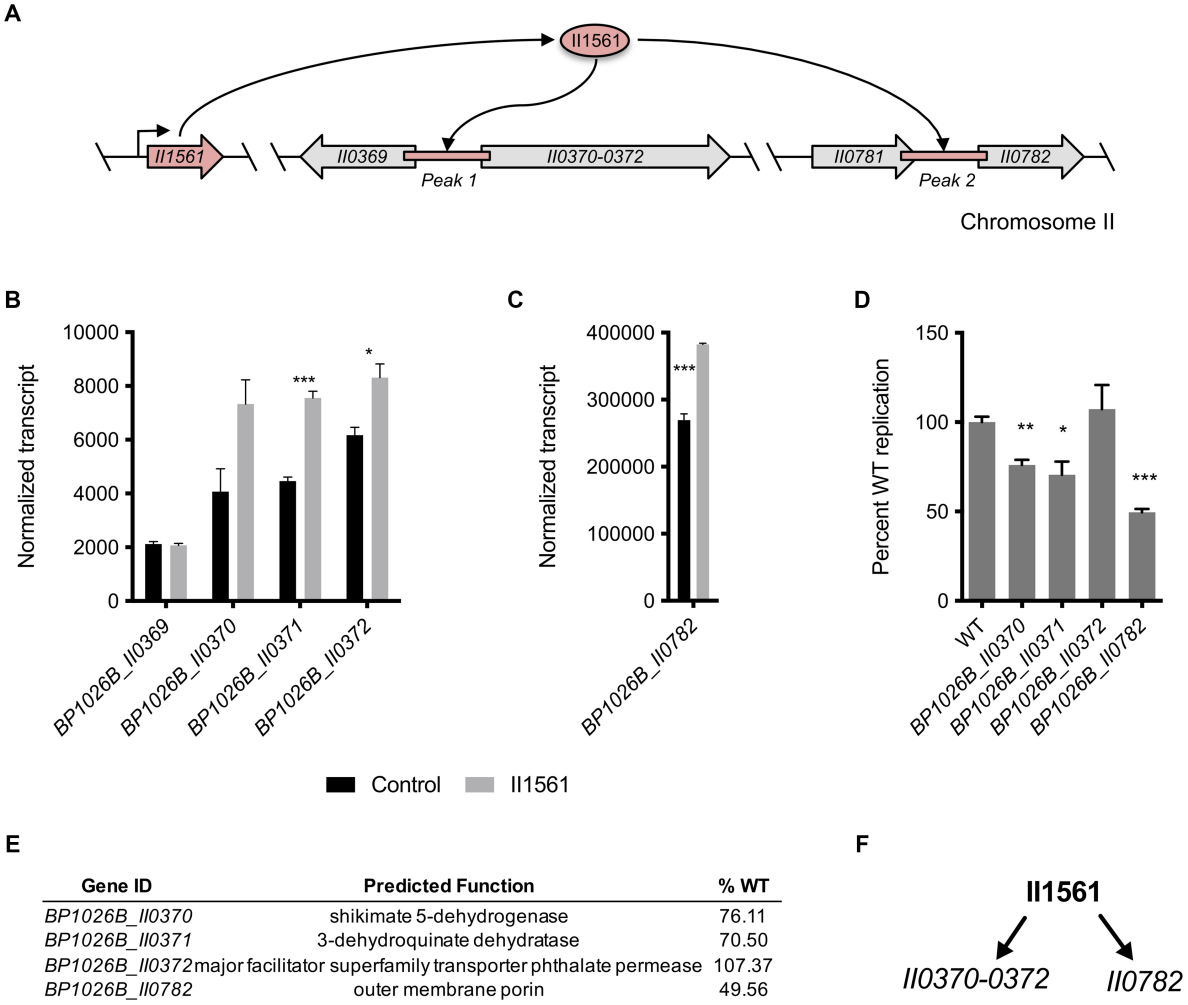
BP1026B_II1561 binds two intergenic regions controlling genes in the shikimate pathway and an outer membrane porin. **(A)** Schematic representation of DNA regions on chromosome II directly bound by BP1026B_II1561. **(B)** Normalized transcript levels of genes surrounding peak 1 comparing the level of transcription in the empty vector control (black bars) and the BP1026B_II1561 expression condition (grey bars). **(C)** Normalized transcript levels of genes surrounding peak 2 from the ChIP-seq data set comparing the level of transcription in the empty vector control (black bars) and the BP1026B_II1561 expression condition (grey bars). **(D)** Transposon mutants were used to infect RAW264.7 cells identifying genes important for intracellular pathogenesis. **(E)** Genes activated by BP1026B_II1561, their predicted functions, and the average wild type replication in RAW264.7 cells (%WT). **(F)** Schematic representation of genes directly controlled by BP1026B_II1561 that contribute to pathogenesis. Arrows represent activation by BP1026B_II1561. Data in bar graphs represent means ± s.e.m. and are analyzed via unpaired *t*-test. *p* values presented are as follows: * < 0.05, ** < 0.01, *** < 0.001.

The BP1026B_II1561 binding peaks overlap intergenic regions between the divergently transcribed genes *BP1026B_II0369* and *BP1026B_II0370*, and between genes *BP1026B_II0781* and *BP1026B_II0782* (Figure 6A). *BP1026B_II0370-II0372* are up-regulated by BP1026B_II1561 with log_2_FCs between 0.58 and 1, while *BP1026B_II0369* shows no change in expression suggesting transcription in a single direction (Figure 6B, Supplementary Table S1). *BP1026B_II0782* is also up-regulated by BP1026B_II1561 with a log_2_FC of 0.52 (Figure 6C, Supplementary Table S1). BP1026B_II0370-II0372 encode shikimate 5-dehydrogenase, 3-dehydroquinate dehydratase, and a phthalate permease, respectively (Figure 6E). BP1026B_II0372 is a major facilitator superfamily phthalate permease that, to our knowledge has not been characterized functionally for its role in pathogenesis. BP1026B_II0370 and BP1026B_II0371 are in the shikimate pathway catalyzing the reactions generating shikimate from 3-dehydroquinate (Kanehisa and Goto, 2000). BP1026B_II0782 is predicted to be an outer-membrane porin that has not been characterized for its role during intracellular infection or general function to our knowledge.

### *BP1026B_II0370, BP1026B_II0371,* and *BP1026B_II0782* are important for *Bp* intracellular survival during RAW264.7 cell infection

We wanted to determine if *BP1026B_II0370-II0372* and *BP1026B_II0782* have a role during intracellular survival because their expression is directly controlled by BP1026B_II1561. Transposon mutants of *BP1026B_II0370-II0372* and *BP1026B_II0782* were used to infect RAW264.7 cells and intracellular bacterial burdens determined at 24 h post infection. A transposon mutant of *BP1026B_II0372* replicated at wild type levels suggesting this gene is dispensable for intracellular infection (Figures 6D,E). Transposon mutants of *BP1026B_II0370* and *BP1026B_II0371* were able to replicate at 76.11 and 70.5% of wild type in the RAW264.7 intracellular environment (Figures 6D,E). We suspect that the pathways using shikimate as an intermediate are responsible for this decrease in intracellular replication. The *BP1026B_II0782* transposon mutant showed the largest defect during RAW264.7 cell infection, replicating at 49.56% of wild type (Figures 6D,E). These transposon mutants maintain identical growth rates as compared to wild type *Bp* 1026b in LB broth indicating that this defect in cellular infection is due to a reduction in RAW264.7 pathogenesis rather than a defect in fitness (Supplementary Figure S3B). The data show that BP1026B_II1561 binds two regions of DNA leading to the up-regulation of four genes, and three of these genes, *BP1026B_II0370, BP1026B_II0371*, and *BP1026B_II0782*, are important for intracellular replication and pathogenesis (Figure 6F).

### Fluorinated tryptophan and anthranilate inhibit *Bp* 1026b growth

**Figure 7:**
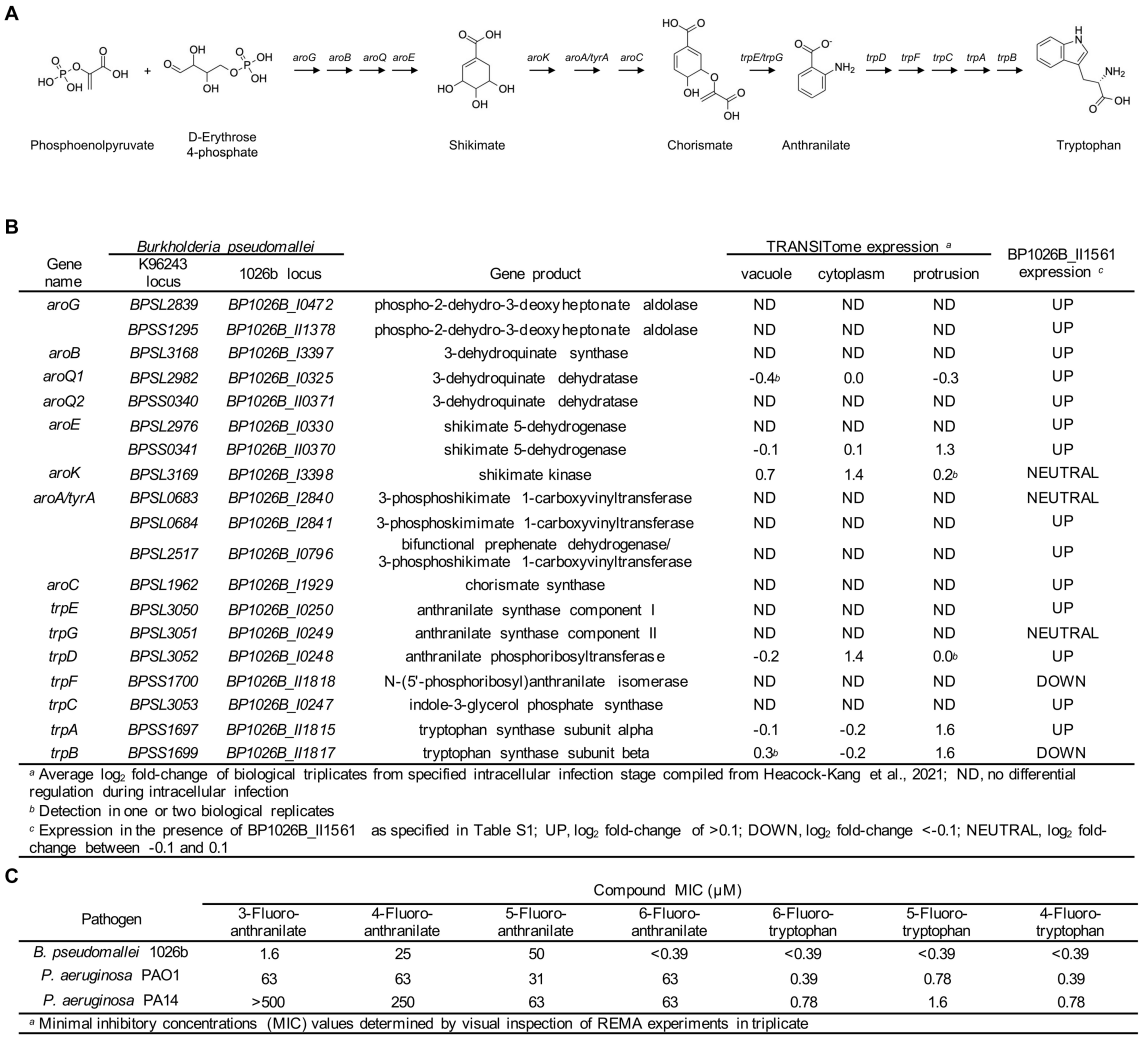
Shikimate and tryptophan biosynthesis pathways in *Bp*. **(A)** Schematic representation of the shikimate and tryptophan biosynthetic pathways is *Bp*. The shikimate pathway converts phosphoenolpyruvate and D-erythrose 4-phosphate into chorismate through a seven step reaction. Chorismate can then be converted into anthranilate via TrpE/G, followed by conversion to tryptophan through five additional steps. **(B)** Genes encoding steps in the shikimate and tryptophan biosynthetic pathways are listed with their gene product. Select genes in these pathways show expression during intracellular transit (vacuole, cytoplasm, and protrusion) (Heacock-Kang et al., 2021) and the majority of these genes are up-regulated in the presence of BP1026B_II1561. **(C)** Various fluorinated analogs of anthranilate and tryptophan show growth inhibition of *Bp* 1026b, *P. aeruginosa* PAO1, and *P. aeruginosa* PA14.

The shikimate pathway generates chorismate that has many downstream products with potential roles in pathogenesis and cell function ranging from aromatic amino acid biosynthesis, biosynthesis of siderophore group nonribosomal peptides, and folate biosynthesis (Kanehisa and Goto, 2000). Downstream of the shikimate pathway, tryptophan is synthesized from chorismate through a biochemically expensive route that includes the intermediate anthranilate (Figure 7A) (Xie et al., 2003). BP1026B_II1561 directly controls the expression of *BP1026B_II0370* and *BP1026B_II0371* that encode enzymes in the shikimate pathway and up-regulates the majority of the shikimate and tryptophan biosynthetic pathways (Figures 7A,B, Supplementary Table S1). Additionally, there is coordinated up-regulation of *BP1026B_II1561*, *BP1026B_II0370, BP1026B_II1815* (*trpA*), and *BP1026B_II1817* (*trpB*) as *Bp* protrudes towards neighboring cells, suggesting that components of both the shikimate and tryptophan biosynthetic pathways are important during intracellular infection (Figures 1A, 7B) (Heacock-Kang et al., 2021). These observations in combination with the critical and diverse roles of tryptophan in other bacterial pathogens (Browne et al., 1970; Zhang and Rubin, 2013; el-Zaatari et al., 2014; Nurul Islam et al., 2019), lead us to hypothesize that these pathways are also important for *Bp* physiology. To test this, we determined the growth inhibition properties of fluorinated tryptophan and anthranilate on *Bp.* Tryptophan with fluorine substitutions at positions 6, 5, and 4 and anthranilate with fluorine substitutions at positions 6, 5, 4, and 3 were used to determine the minimal inhibitory concentration (MIC) in minimal media. Growth of *Bp* was significantly inhibited by 6-fluorotryptohan, 5-fluorotryptohan, 4-fluorotryptohan, and 6-fluoroanthranilate with MICs <0.39 μM (Figure 7C). Inhibition of *Bp* was also observed in the presence of 3-fluoroanthranilate with an MIC of 1.6 μM while 4-fluoroanthranilate and 5-fluoroanthranilate showed MICs of 25 μM and 50 μM (Figure 7C). A similar trend was observed with other respiratory pathogens including *P. aeruginosa* PAO1 and *P. aeruginosa* PA14. In all cases, the position of fluorine on tryptophan had a minute influence on the level of inhibition with MICs ranging from 0.39 μM to 1.6 μM (Figure 7C). *P. aeruginosa* strains were less inhibited by fluoroanthranilate compounds with MICs ranging from 31 μM to >500 μM (Figure 7C). Although *Bp*, and the other respiratory pathogens tested, are not tryptophan auxotrophs, the incorporation of fluorine into tryptophan and/or anthranilate show significant growth inhibition. These data, taken together with the expression of shikimate and tryptophan biosynthetic enzymes by BP1026B_II1561 and during *Bp* intracellular survival, suggest that these pathways/enzymes may be targets for novel therapeutic interventions against *Bp* infection.

## Discussion

*Bp* encounters many environmental niches during its existence in the soil, in association with the rhizosphere, and during infection of a host. In the host, *Bp* must navigate another complex set of environments including invasion of the host cell, escape of the vacuole, cytoplasmic replication, and spread towards neighboring cells all while avoiding host cell defenses (Heacock-Kang et al., 2021). For *Bp* to be successful in all of these unique environments, it must encode a large number of regulators to control pathways and mechanisms for survival. Previously, we have shown that 1,953 genes are differentially regulated during the intracellular lifecycle of *Bp,* further highlighting the importance of a sophisticated system of regulation during infection (Heacock-Kang et al., 2021). BP1026B_II1561 was identified to be up-regulated as *Bp* protrudes towards neighboring cells, indicating a role during later stages of intracellular infection. BP1026B_II1561 is critical for complete pathogenesis in RAW264.7 cell infection and BALB/c mouse infection leading us to investigate the transcriptional role of this regulator. The primary sequence of BP1026B_II1561 indicates it is similar to a TetR family transcriptional regulator. This family of regulators is classically thought to control antibiotic resistance and small-molecule export but has been tied to many other functions including metabolism, antibiotic production, and quorum sensing (Cuthbertson and Nodwell, 2013). For this reason, we took a broad approach to study the role of BP1026B_II1561 through the identification of the regulation network and partial characterization of this network in pathogenesis. In general, we see that the expression of BP1026B_II1561 leads to variation in the expression of many known metabolic pathways and a large number of hypothetical genes that could be important for intracellular survival and pathogenesis.

Modulation of secondary metabolite expression includes the up-regulation of the siderophores malleobactin (Alice et al., 2006), malleilactone (Biggins et al., 2012), and pyochelin (Biggins et al., 2014), by BP1026B_II1561, suggesting a role in iron scavenging during intracellular infection. Although our data indicate that the expression of these siderophores contributes to pathogenesis in BALB/c mice, previous studies have shown that some of these factors are dispensable (Kvitko et al., 2012). In addition, the down-regulation of malleipeptin (Biggins et al., 2014) by BP1026B_II1561 is curious, as malleipeptin is required for pathogenesis in BALB/c mice. However, the suggestion that malleipeptin is required for invasion of the epithelium fits with our data as BP1026B_II1561 is up-regulated after invasion during protrusion. In this case, BP1026B_II1561-dependent down regulation of malleipeptin could be an energy saving mechanism when there is no longer a need for this virulence-associated secondary metabolite. Considering this data together, we start to see a complex picture of the BP1026B_II1561 regulation network where many factors are controlled for various purposes at different stages of infection.

Genes involved in propanoate and fatty acid metabolism are down-regulated by BP1026B_II1561 to potentially conserve or shift metabolic energy during intracellular survival. During infection, *Bp* must rely on nutrients obtained from the host cell to replicate and survive. When nutrients are depleted, *Bp* will protrude towards neighboring cells to form MNGCs and gain access to additional nutrients. BP1026B_II1561 is up-regulated during protrusion and down-regulates genes that direct intermediaries into energy producing molecules like acetyl-CoA through various pathways. The energy saved could allow proper function of other virulence factors like T6SS-1, which is used for fusing host cells into MNGCs thereby gaining access to nutrients (French et al., 2011). Transposon mutants of the genes involved in fatty acid and propanoate metabolism showed reduced intracellular replication at various levels indicating that they are important for pathogenesis and that down-regulating energy production is critical for full infection. BP1026B_II0232 is an accessory protein for AcnD causing *trans* to *cis* isomerization of methyl aconitate (Garvey et al., 2007) and its mutant replicates at 26.25% of wild type while not showing any defect during *in vitro* growth. Further investigation into this gene and its function could elucidate a better understanding of this metabolic pathway during pathogenesis.

Another potential regulatory role of BP1026B_II1561 is observed in the up-regulation of BP1026B_I0071 (PrtR), a potential transmembrane regulator. PrtR is a predicted two-component regulatory system that functions with BP1026B_I0072 (PrtI), an ECF sigma factor. The PrtIR regulatory complex has been tied to several functions including expression of the AprX extracellular protease in *Pseudomonas fluorescens* LS107d2 (Burger et al., 2000), protease production in *P. entomophila* (Liehl et al., 2006), protease/cycliclipopetide production in *P. fluorescens* strains HCl-07 and SS101 (Song et al., 2014; Yang et al., 2014), and germination arrest factor (GAF) by *P. fluorescens* WH6 (Okrent et al., 2014). GAF is a secondary metabolite that has been tied to herbicidal and antibacterial functions in *P. fluorescens* WH6, a rhizosphere associated bacteria (Okrent et al., 2014). Recognizing that *Bp* is also a rhizosphere-associated organism, the role of *prtR* in *Bp* could be similar to *P. fluorescens*. In the same transcriptional snapshot where we see up-regulation of *prtR*, we see differential expression of various secondary metabolites that are involved in iron acquisition and virulence. Replication of the *prtR* transposon mutant was significantly reduced during intracellular infection of RAW264.7 cells indicating that this regulatory pathway is important for pathogenesis. These downstream virulence functions of *prtR* could carry over from the normal *Bp* lifecycle within the rhizosphere, but still have an effect on mammalian cells. Furthermore, this highlights the complexity of the BP1026B_II1561 regulation network and its role during infection.

We have shown previously that glyphosate, acting as an antibacterial agent blocking the shikimate pathway, can severely inhibits *Bp* replication (Norris et al., 2009). This highlights how critically essential the shikimate pathway is for *Bp* replication and survival. BP1026B_II1561 up-regulates several genes in the shikimate pathway including 3-dehydroquinate dehydratase (BP1026B_II0371) and shikimate 5-dehydrogenase (BP1026B_II0370). The enzymes encoded by these genes catalyze two steps in the reaction from phosphoenolpyruvate and D-erythrose 4-phosphate to chorismate (Figure 7A), which can be shunted towards various pathways including aromatic amino acid and folate biosynthesis (Bentley, 1990; Kanehisa and Goto, 2000). The shikimate pathway is absent from mammals marking this as a potential target for the development of novel chemotherapeutic interventions (Roberts et al., 1998; Herrmann and Weaver, 1999; Coggins et al., 2003; Ducati et al., 2007; Mir et al., 2015; Nunes et al., 2020). BP1026B_II1561 induces slight up-regulation of the majority of genes encoding enzymes in the shikimate and tryptophan biosynthetic pathways as well (Figure 7B). Coordinated expression of *BP1026B_II1561, BP1026B_II1815* (*trpA*), and *BP1026B_II1817* (*trpB*) as *Bp* protrudes towards neighboring host cells suggests that tryptophan biosynthesis is also important during intracellular infection (Heacock-Kang et al., 2021). Furthermore, inhibition of *Bp* growth by fluorotryptophan analogs and some fluoroanthranilate analogs further indicates these pathways/enzymes as potential drug targets. Because *Bp* is not an auxotroph for tryptophan and contains a complete biosynthetic pathway, the precise mechanism for fluorotryptophan growth inhibition of *Bp* is unknown, but could be due to cytotoxic effects from the incorporation of flourotryptophan into proteins as observed in *Escherichia coli* (Browne et al., 1970). However, this highlights the lack of information on *Bp* tryptophan biosynthesis in the context of intracellular infection. Tryptophan is a critical growth component that can be limited by the host cell to defend against infection. Indoleamine-2,3-dioxygenase (IDO) sequesters host cell tryptophan by converting it to kynurenine (Hayashi et al., 2001; Silva et al., 2002). While *Mycobacterium tuberculosis* is able to counteract the effects of IDO expression through tryptophan biosynthesis (Zhang and Rubin, 2013), it is still partially susceptible to growth inhibition of fluorotryptophan and fluorinated intermediates of tryptophan biosynthesis (Nurul Islam et al., 2019). BP1026B_II1561 up-regulation of the majority of enzymes in the shikimate and tryptophan biosynthetic pathways, the coordinated expression of several of these enzymes during intracellular infection, and the nanomolar level of growth inhibition by fluorotryptophan analogs indicate that these pathways/enzymes could be a rational target for the development of novel chemotherapeutic interventions against *Bp* infection.

Overall, the transcriptional regulator BP1026B_II1561 plays a critical role during pathogenesis, marked by attenuation of its mutant in cell culture and BALB/c mice. BP1026B_II1561 has a broad effect on the transcription of many pathways and genes that are involved in pathogenesis by way of metabolic function. Future analysis of these pathways, particularly shikimate and tryptophan biosynthesis, will shed light on the metabolic requirements and further describe the pathogenesis of *Bp* during intracellular infection. Although the BP1026B_II1561 regulation network is only one part of the *Bp* pathogenic regulon, it is nonetheless complex and plays a critical role in the sophisticated intracellular lifecycle of *Bp*.

## Methods and materials

### Bacterial strains, media, and culture conditions

*Escherichia coli* strain EPMax10B (BioRad), E1869, and E1354 were used for cloning or plasmid mobilization into *Bp* as described previously (Kang et al., 2009; Norris et al., 2009). Luria-Bertani (LB) medium (Difco) or 1x M9 minimal medium supplemented with 20 mM glucose (MG) were used to culture all strains. MG media was supplemented with 0.3% (v/v) glyphosate when appropriate. The select agent excluded analog of *Bp* 1026b, *Bp*82, was used where appropriate (Propst et al., 2010). All manipulation and experiments involving *Bp* 1026b were conducted in a CDC-approved and registered facility at the University of Hawaiʻi at Mānoa or Colorado State University with prior approval by internal review and adherence to recommendations set forth in the BMBL, 5^th^ edition (Wilson and Chosewood, 2007) for BSL3 organisms.

### Molecular methods and reagents

Molecular methods and reagents were used as described previously (Norris et al., 2009, 2010, 2011; Kang et al., 2011). An in-frame deletion mutant of BP1026B_II1561 was generated using lambda-red recombineering with minor modification (Kang et al., 2011). Lambda red genes were PCR amplified from pKaKa1 and co-incubated with the knockout fragment of BP1026B_II1561 and mutants selected on glyphosate. For expression of BP1026B_II1561, BP1026B_II1561 was PCR amplified with oligos 2,660 (5′- AAT GCG CCG CAT ATG CCG CCG TCC GAT CAC GCC AAA ATG) and 2,661 (5′- ATG TCG AGC TCG ACG CCG ATG CCG) digested with *Nde*I and *Sac*I and ligated into *Nde*I/*Sac*I-cut pAM3GIQ-3xTY1. Confirmation of pAM3GIQ-3xTY1-BP1026B_II1561 was done by *Afl*II/*Sac*I, *Nde*I, and *Sal*I digests, sequencing, and expression of BP1026B_II1561 was confirmed via western blot analysis.

### Growth analysis

All strains were first grown overnight in LB broth at 37°C, harvested the following day, diluted to an OD_600_ of 0.1 in 200 μL of fresh LB, and placed into 96-well plates in duplicate. Growth curves were carried out at 37°C with shaking, data was recorded with the BioTek ELx808IU plate reader, and measurements taken at an OD_630_ every 30 min.

### Cell infection assays

Intracellular replication and MNGC formation assays were carried out as previously described (Heacock-Kang et al., 2018a,b,c) with minor modifications. Briefly, RAW264.7 murine macrophages were seeded for infection at 80–90% confluence on Corning CellBIND culture plates, allowed to attach overnight, washed twice with 1XPBS, and infected the following day. For intracellular replication assays, *Bp* 1026b, the BP1026B_II1561 mutant, or the BP1026B_II1561 complement (mutant with pAM3GIQ-3xTY1-BP1026B_II1561) were allowed to infect monolayers at an MOI of 1:1, washed with 1XPBS, and then DMEM supplemented with 10% (v/v) FBS, 0.1 mM IPTG to drive expression of the complement, 700 μg/mL amikacin and 700 μg/mL kanamycin were added to kill any extracellular bacteria. At 2, 8, and 24 h post-infection, infected monolayers were lysed with 0.2% (v/v) Triton X-100. Serial dilutions of lysates were plated on LB and colony-forming units (CFU) per well were determined. MNGC formation assays were carried out as intracellular replication assays, with the exception that 1.2% (w/v) low-melt agarose was added to DMEM after infection allowing the formation of MNGCs. At 24 h post-infection, monolayers were fixed with 4% (w/v) paraformaldehyde in PBS, agarose plugs removed, stained with 0.05% crystal violet, and MNGC diameters measured with the Zeiss Axio Observer D1 and the AxioVision 64 bit 4.9.1 software. Cell infection assays with mutants from the *Bp* 1026b::T24 transposon mutant library were carried out as described above although kanamycin was removed and amikacin added at a final concentration of 1,500 μg/mL to remove extracellular bacteria post infection. Monolayers were lysed at 24 h post-infection as described above. All transposon mutants tested were confirmed to be in the gene of interest by sequencing. Percent wild type infection (%WT) was calculated with the following formula: %WT = (CFU_mutant_/mean CFU_WT_) x 100.

### Animal studies

BALB/c mice between 4 and 6 weeks of age were purchased from Charles River Laboratory. All infections with *Bp* strains (wild type and the BP1026B_II1561 mutant) were administered via the intranasal (i.n.) inoculation route. Mice were anesthetized with 100 mg/kg of ketamine plus 10 mg/kg of xylazine. The challenge dose (4,500 CFU) of each *Bp* strain was suspended in 20 μL of 1XPBS and used to inoculate each mouse via the i.n. route. Each strain was used to inoculate 5 mice. Animals were monitored for disease symptoms daily and euthanized at predetermined humane endpoints. The lungs, livers, and spleens of surviving mice were harvested, homogenized, serially diluted, and plated on LB to determine bacterial burdens. Survival characteristics were plotted using Prism software (GraphPad, La Jolla, CA) and statistical analysis was done by Kaplan–Meier curves.

### RNA-seq and ChIP-seq analysis

RNA-seq and ChIP-seq analysis were carried out under the same conditions. Briefly, *Bp*82 expressing BP1026B_II1561 from pAM3GIQ-3xTY1-*BP1026B_II1561* was grown overnight and sub-cultured to mid-log phase in LB + adenine+0.1 mM IPTG in triplicate. An empty vector (pAM3GIQ-3xTY1) was used as a control. Total RNA was harvested using RNeasy Mini Kit (Qiagen) with on-column (Qiagen) and off-column (Epicentre) DNase digestion steps. RNA samples were sent to Tufts University Genomics Core (TUCF Genomics) for library preparation and Illumina 50 bp single-end reads were sequenced on the Illumina HiSeq 2,500. RNA-seq data were analyzed with Rockhopper (McClure et al., 2013).

ChIP-seq was carried out as previously described with minor modifications (Heacock-Kang et al., 2018a,b,c). Briefly, ChIP-seq samples were grown identically as RNA-seq samples, harvested, and fixed with 4% (w/v) paraformaldehyde in PBS, followed by shearing of DNA-protein complexes with the Covaris M220 ultrafocused sonicator. Cell debris was removed and DNA-protein complexes were immunoprecipitated with anti-TY1-tag monoclonal antibody (Diagenode C15200054) and secondary antibodies conjugated to magnetic beads (Diagenode C03010022). DNA-protein complexes were washed, decrosslinked, treated with RNaseA and proteinase K, and purified with QIAquick PCR purification kit. Immunoprecipitated DNA was sent to TUCF Genomics where DNA libraries were prepped and 50 bp single-end reads were sequenced with the Illumina HiSeq 2,500. ChIP-seq data was aligned to the *Bp* 1026b genome with Bowtie2 (Langmead and Salzberg, 2012), peaks called with MACS2 (Zhang et al., 2008), and consensus binding regions determined with MEME (Bailey and Elkan, 1994).

### Determination of MICs for anthranilate and tryptophan analogs

Overnight cultures of *Bp* 1026b, *P. aeruginosa* PAO1, and *P. aeruginosa* PA14 were grown in LB and diluted 1:30 into minimal medium (MM) and incubated in a 37°C shaking incubator at 250 rpm until the OD_600_ reached 0.7. Vogel Bonner Minimal Medium (VBMM) and M9 minimal medium were used for cultivation of *P. aeruginosa* and *Burkholderia* spp., respectively. *P. aeruginosa* PAO1 and *P. aeruginosa* PA14 were chosen for comparison because they are well established Gram-negative pathogens that are intrinsically resistant to antimicrobials. Cultures were diluted to a final OD_600_ of 0.1 in a sterile saline solution (0.85% (w/v) NaCl) and 200 μL was added to 9.8 mL MM. The culture solution was mixed with an 11 mg/mL resazurin (Sigma) solution at a dilution of 20 μL of resazurin (also known as alamar blue) solution per ml. A 50 μL aliquot of mixed solution was added to each well of a 96-well plate containing 50 μL of each compound diluted in MM at the defined concentrations. The microtiter plate was then statically incubated for 24 h at 37°C. The color of the resazurin indicator was used to identify inhibitory concentrations of anthranilate and tryptophan analogs (Sigma). Fluorinated tryptophan and anthranilate analogs have molecular weights of 222.22 and 155.13, respectively.

## Data availability statement

The datasets presented in this study can be found in online repositories. The names of the repository/repositories and accession number(s) can be found at: https://www.ncbi.nlm.nih.gov/geo/, GSE273459 and GSE273460.

## Ethics statement

The animal study was approved by Institutional Animal Care and Use Committee at the University of Hawaiʻi at Mānoa. The study was conducted in accordance with the local legislation and institutional requirements.

## Author contributions

IM: Writing – original draft, Writing – review & editing, Conceptualization, Investigation, Formal analysis. MN: Writing – review & editing, Conceptualization, Investigation, Formal analysis. YH-K: Writing – review & editing, Conceptualization, Investigation. JZ-S: Writing – review & editing, Investigation. ZS: Writing – review & editing, Investigation. BH: Writing – review & editing, Investigation, Formal analysis. LF: Writing – review & editing, Investigation, Formal analysis. MI: Writing – review & editing, Conceptualization, Resources. DC: Writing – review & editing, Conceptualization, Resources. BB: Writing – review & editing, Conceptualization, Resources. TH: Writing – review & editing, Conceptualization, Resources, Funding acquisition.

## Supplementary material

SUPPLEMENTARY FIGURE S1 WoPPER analysis reveals 40 gene clusters regulated by BP1026B_II1561 on *Bp* 1026b chromosome I. **(A)** Circular map of *Bp* 1026b chromosome I showing up-regulated (yellow) and down-regulated (blue) gene clusters of BP1026B_II1561. The outer circle is the plus strand (dark grey) and the inner circle is the negative strand (light grey). **(B)** Linear map of BP1026B_II1561 regulated gene clusters showing the mean log_2_FC of each cluster versus its position on chromosome I. The orange line represents the plus strand and the blue line represents the negative strand. The size of each gene cluster circle represents the number of genes within that given cluster. **(C)** Table summarizing the gene clusters regulated by BP1026B_II1561.

SUPPLEMENTARY FIGURE S2 WoPPER analysis reveals 50 gene clusters regulated by BP1026B_II1561 on *Bp* 1026b chromosome II. **(A)** Circular map of *Bp* 1026b chromosome II showing up-regulated (yellow) and down-regulated (blue) gene clusters of BP1026B_II1561. The outer circle is the plus strand (dark grey) and the inner circle is the negative strand (light grey). **(B)** Linear map of BP1026B_II1561 regulated gene clusters showing the mean log_2_FC of each cluster versus its position on chromosome II. The orange line represents the plus strand and the blue line represents the negative strand. The size of each gene cluster circle represents the number of genes within that given cluster. **(C)** Table summarizing the gene clusters regulated by BP1026B_II1561 on chromosome II.

SUPPLEMENTARY FIGURE S3 Growth analysis of transposon mutants compared to wild type *Bp* 1026b. **(A)** Mutants indirectly controlled by BP1026B_II1561 that show defects during intracellular replication (Figures 4B,C) were tested for their ability to grow in LB. All mutants showed identical growth patterns to wild type *Bp* 1026b (WT) indicating that the defects during intracellular infection were not due to a defect in fitness. **(B)** A BP1026B_II0782 showed a significant defect during intracellular pathogenesis (Figures 6D,E) and shows no defect in fitness.
