# Supplementary material for: TetR-like regulator BP1026B_II1561 controls aromatic amino acid biosynthesis and intracellular pathogenesis in Burkholderia pseudomallei
Source: Front Microbiol. 2024 Aug 15;15:1441330. doi: 10.3389/fmicb.2024.1441330 (PMC11358695; doi:10.3389/fmicb.2024.1441330)
Supplement: SUPPLEMENTARY FIGURE S1 — WoPPER analysis reveals 40 gene clusters regulated by BP1026B_II1561 on Bp 1026b chromosome I. (A) Circular map of Bp 1026b chromosome I showing up-regulated (yellow) and down-regulated (blue) gene clusters of BP1026B_II1561. The outer circle is the plus strand (dark grey) and the inner circle is the negative strand (light grey). (B) Linear map of BP1026B_II1561 regulated gene clusters showing the mean log2FC of each cluster versus its position on chromosome I. The orange line represents the plus strand and the blue line represents the negative strand. The size of each gene cluster circle represents the number of genes within that given cluster. (C) Table summarizing the gene clusters regulated by BP1026B_II1561. [file Image_1.PDF]

a

b

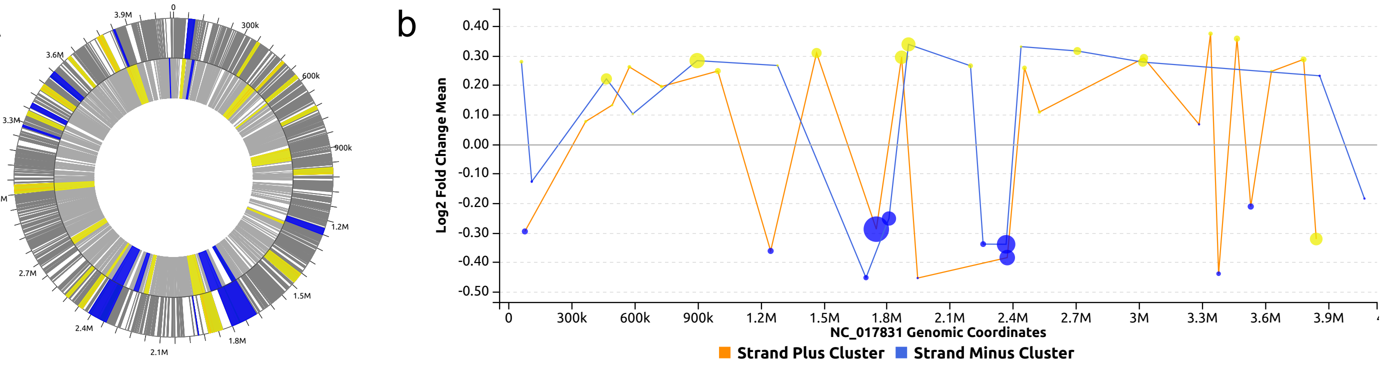

c

Cluster	Start Coordinates	End Coordinates	Strand	Gene ID	Expression
1	50668	66682	-	BP1026B_I0048;BP1026B_I0049;BP1026B_I0050;BP1026B_I0051;BP1026B_I0052;BP1026B_I0053;BP1026B_I0054;BP1026B_I0055;BP1026B_I0056;BP1026B_I0057;BP1026B_I0060;BP1026B_I0061;BP1026B_I0062	UP
2	61805	88125	+	BP1026B_I0059;BP1026B_I0058;BP1026B_I0063;BP1026B_I0064;BP1026B_I0065;BP1026B_I0066;BP1026B_I0069;BP1026B_I0070;BP1026B_I0073;BP1026B_I0074;BP1026B_I0078;BP1026B_I0079;BP1026B_I0080	DOWN
3	103502	114477	-	BP1026B_I0097;BP1026B_I0098;BP1026B_I0099;BP1026B_I0100;BP1026B_I0106;BP1026B_I0107;BP1026B_I0108	DOWN
4	359756	372710	+	BP1026B_I0351;BP1026B_I0352;BP1026B_I0356;BP1026B_I0357;BP1026B_I0361;BP1026B_I0364	UP
5	438386	487029	-	BP1026B_I0428;BP1026B_I0429;BP1026B_I0430;BP1026B_I0431;BP1026B_I0432;BP1026B_I0433;BP1026B_I0434;BP1026B_I0439;BP1026B_I0440;BP1026B_I0441;BP1026B_I0442;BP1026B_I0443;BP1026B_I0451;BP1026B_I0452;BP1026B_I0453;BP1026B_I0464;BP1026B_I0465;BP1026B_I0466;BP1026B_I0467;BP1026B_I0468;BP1026B_I0469	UP
6	489186	499751	+	BP1026B_I0471;BP1026B_I0476	UP
7	567168	583497	+	BP1026B_I0529;BP1026B_I0530;BP1026B_I0531;BP1026B_I0532;BP1026B_I0533;BP1026B_I0534;BP1026B_I0535;BP1026B_I0536;BP1026B_I0537;BP1026B_I0539;BP1026B_I0538;BP1026B_I0541;BP1026B_I0540;BP1026B_I0542;BP1026B_I0544;BP1026B_I0545	UP
8	584761	590384	-	BP1026B_I0547;BP1026B_I0548;BP1026B_I0549;BP1026B_I0550;BP1026B_I0551;BP1026B_I0552;BP1026B_I0553;BP1026B_I0554	UP
9	718317	733526	+	BP1026B_I0669;BP1026B_I0674;BP1026B_I0675;BP1026B_I0676;BP1026B_I0679;BP1026B_I0680;BP1026B_I0683;BP1026B_I0682;BP1026B_I0687;BP1026B_I0686	UP
10	861212	927706	-	BP1026B_I0807;BP1026B_I0809;BP1026B_I0816;BP1026B_I0819;BP1026B_I0820;BP1026B_I0823;BP1026B_I0825;BP1026B_I0827;BP1026B_I0828;BP1026B_I0829;BP1026B_I0831;BP1026B_I0832;BP1026B_I0834;BP1026B_I0835;BP1026B_I0836;BP1026B_I0837;BP1026B_I0840;BP1026B_I0841;BP1026B_I0844;BP1026B_I0845;BP1026B_I0846;BP1026B_I0848;BP1026B_I0849;BP1026B_I0852;BP1026B_I0858;BP1026B_I0859;BP1026B_I0860;BP1026B_I0861;BP1026B_I0862;BP1026B_I0863;BP1026B_I0869;BP1026B_I0871	UP
11	981465	1007411	+	BP1026B_I0924;BP1026B_I0925;BP1026B_I0926;BP1026B_I0927;BP1026B_I0928;BP1026B_I0930;BP1026B_I0929;BP1026B_I0931;BP1026B_I0932;BP1026B_I0934;BP1026B_I0935;BP1026B_I0936;BP1026B_I0937;BP1026B_I0938;BP1026B_I0939;BP1026B_I0941;BP1026B_I0948;BP1026B_I0950	UP
12	1234833	1261337	+	BP1026B_I1158;BP1026B_I1159;BP1026B_I1160;BP1026B_I1161;BP1026B_I1162;BP1026B_I1163;BP1026B_I1164;BP1026B_I1165;BP1026B_I1166;BP1026B_I1167;BP1026B_I1168;BP1026B_I1169;BP1026B_I1170;BP1026B_I1171;BP1026B_I1173;BP1026B_I1172;BP1026B_I1174;BP1026B_I1175	DOWN
13	1272459	1281740	-	BP1026B_I1184;BP1026B_I1187;BP1026B_I1193	UP
14	1444205	1488349	+	BP1026B_I1342;BP1026B_I1344;BP1026B_I1347;BP1026B_I1351;BP1026B_I1352;BP1026B_I1358;BP1026B_I1359;BP1026B_I1360;BP1026B_I1361;BP1026B_I1363;BP1026B_I1364;BP1026B_I1365;BP1026B_I1366;BP1026B_I1367;BP1026B_I1370;BP1026B_I1371;BP1026B_I1372;BP1026B_I1373;BP1026B_I1374;BP1026B_I1375;BP1026B_I1376;BP1026B_I1377;BP1026B_I1378;BP1026B_I1379;BP1026B_I1383	UP
15	1689391	1712173	-	BP1026B_I1565;BP1026B_I1568;BP1026B_I1582;BP1026B_I1584	DOWN
16	1690753	1801496	+	BP1026B_I1567;BP1026B_I1569;BP1026B_I1570;BP1026B_I1571;BP1026B_I1572;BP1026B_I1573;BP1026B_I1574;BP1026B_I1575;BP1026B_I1576;BP1026B_I1577;BP1026B_I1578;BP1026B_I1579;BP1026B_I1580;BP1026B_I1581;BP1026B_I1583;BP1026B_I1585;BP1026B_I1587;BP1026B_I1588;BP1026B_I1589;BP1026B_I1590;BP1026B_I1591;BP1026B_I1592;BP1026B_I1593;BP1026B_I1594;BP1026B_I1595;BP1026B_I1596;BP1026B_I1597;BP1026B_I1598;BP1026B_I1599;BP1026B_I1600;BP1026B_I1601;BP1026B_I1602;BP1026B_I1603;BP1026B_I1604;BP1026B_I1605;BP1026B_I1606;BP1026B_I1607;BP1026B_I1608;BP1026B_I1609;BP1026B_I1610;BP1026B_I1611;BP1026B_I1612;BP1026B_I1613;BP1026B_I1617;BP1026B_I1620;BP1026B_I1621;BP1026B_I1622;BP1026B_I1630;BP1026B_I1631;BP1026B_I1632;BP1026B_I1635;BP1026B_I1636;BP1026B_I1642;BP1026B_I1643;BP1026B_I1645;BP1026B_I1647;BP1026B_I1648;BP1026B_I1652;BP1026B_I1653;BP1026B_I1654;BP1026B_I1655;BP1026B_I1656;BP1026B_I1657;BP1026B_I1659;BP1026B_I1660;BP1026B_I1661;BP1026B_I1662	DOWN
17	1775393	1837548	-	BP1026B_I1637;BP1026B_I1638;BP1026B_I1639;BP1026B_I1640;BP1026B_I1641;BP1026B_I1644;BP1026B_I1646;BP1026B_I1649;BP1026B_I1650;BP1026B_I1651;BP1026B_I1658;BP1026B_I1663;BP1026B_I1664;BP1026B_I1665;BP1026B_I1666;BP1026B_I1667;BP1026B_I1668;BP1026B_I1669;BP1026B_I1670;BP1026B_I1671;BP1026B_I1672;BP1026B_I1673;BP1026B_I1674;BP1026B_I1675;BP1026B_I1676	DOWN
18	1842173	1900708	+	BP1026B_I1681;BP1026B_I1683;BP1026B_I1684;BP1026B_I1687;BP1026B_I1689;BP1026B_I1690;BP1026B_I1693;BP1026B_I1692;BP1026B_I1694;BP1026B_I1695;BP1026B_I1696;BP1026B_I1697;BP1026B_I1698;BP1026B_I1699;BP1026B_I1700;BP1026B_I1701;BP1026B_I1702;BP1026B_I1705;BP1026B_I1707;BP1026B_I1709;BP1026B_I1710;BP1026B_I1714;BP1026B_I1716;BP1026B_I1717;BP1026B_I1718;BP1026B_I1719;BP1026B_I1720;BP1026B_I1721;BP1026B_I1728;BP1026B_I1729;BP1026B_I1730	UP
19	1872678	1933803	-	BP1026B_I1708;BP1026B_I1711;BP1026B_I1712;BP1026B_I1713;BP1026B_I1715;BP1026B_I1722;BP1026B_I1723;BP1026B_I1724;BP1026B_I1725;BP1026B_I1726;BP1026B_I1727;BP1026B_I1731;BP1026B_I1732;BP1026B_I1733;BP1026B_I1734;BP1026B_I1735;BP1026B_I1738;BP1026B_I1739;BP1026B_I1740;BP1026B_I1741;BP1026B_I1742;BP1026B_I1743;BP1026B_I1744;BP1026B_I1745	UP
20	1942190	1944000	+	BP1026B_I1751;BP1026B_I1752;BP1026B_I1753;BP1026B_I1754	DOWN
21	2186981	2208393	-	BP1026B_I1967;BP1026B_I1968;BP1026B_I1969;BP1026B_I1970;BP1026B_I1971;BP1026B_I1973;BP1026B_I1975;BP1026B_I1976;BP1026B_I1978;BP1026B_I1981;BP1026B_I1982;BP1026B_I1983	UP
22	2242307	2267593	-	BP1026B_I2008;BP1026B_I2009;BP1026B_I2011;BP1026B_I2013;BP1026B_I2014;BP1026B_I2015;BP1026B_I2016;BP1026B_I2017;BP1026B_I2018;BP1026B_I2019;BP1026B_I2020;BP1026B_I2021;BP1026B_I2022;BP1026B_I2023;BP1026B_I2024;BP1026B_I2025;BP1026B_I2026;BP1026B_I2027;BP1026B_I2028;BP1026B_I2029;BP1026B_I2030;BP1026B_I2031	DOWN
23	2330918	2411419	+	BP1026B_I2079;BP1026B_I2081;BP1026B_I2086;BP1026B_I2117;BP1026B_I2118;BP1026B_I2123;BP1026B_I2133;BP1026B_I2136;BP1026B_I2139;BP1026B_I2141;BP1026B_I2142;BP1026B_I2143;BP1026B_I2146;BP1026B_I2148;BP1026B_I2149;BP1026B_I2150;BP1026B_I2151;BP1026B_I2152;BP1026B_I2153;BP1026B_I2154;BP1026B_I2155;BP1026B_I2156;BP1026B_I2160;BP1026B_I2161;BP1026B_I2163;BP1026B_I2165;BP1026B_I2169;BP1026B_I2171;BP1026B_I2179;BP1026B_I2184	DOWN
24	2331963	2399355	-	BP1026B_I2080;BP1026B_I2082;BP1026B_I2083;BP1026B_I2085;BP1026B_I2087;BP1026B_I2088;BP1026B_I2089;BP1026B_I2090;BP1026B_I2091;BP1026B_I2092;BP1026B_I2093;BP1026B_I2094;BP1026B_I2095;BP1026B_I2096;BP1026B_I2097;BP1026B_I2098;BP1026B_I2099;BP1026B_I2100;BP1026B_I2101;BP1026B_I2102;BP1026B_I2103;BP1026B_I2104;BP1026B_I2105;BP1026B_I2106;BP1026B_I2107;BP1026B_I2108;BP1026B_I2109;BP1026B_I2110;BP1026B_I2111;BP1026B_I2112;BP1026B_I2113;BP1026B_I2114;BP1026B_I2115;BP1026B_I2116;BP1026B_I2119;BP1026B_I2120;BP1026B_I2121;BP1026B_I2122;BP1026B_I2124;BP1026B_I2125;BP1026B_I2126;BP1026B_I2127;BP1026B_I2128;BP1026B_I2129;BP1026B_I2130;BP1026B_I2131;BP1026B_I2132;BP1026B_I2134;BP1026B_I2135;BP1026B_I2137;BP1026B_I2138;BP1026B_I2140;BP1026B_I2144;BP1026B_I2145;BP1026B_I2157;BP1026B_I2158;BP1026B_I2159;BP1026B_I2167;BP1026B_I2168;BP1026B_I2170;BP1026B_I2172	DOWN
25	2430474	2441381	-	BP1026B_I2201;BP1026B_I2202;BP1026B_I2203;BP1026B_I2204;BP1026B_I2205;BP1026B_I2206;BP1026B_I2207;BP1026B_I2208;BP1026B_I2209;BP1026B_I2210;BP1026B_I2211;BP1026B_I2212	UP
26	2442278	2462823	+	BP1026B_I2213;BP1026B_I2216;BP1026B_I2218;BP1026B_I2219;BP1026B_I2221;BP1026B_I2222;BP1026B_I2223;BP1026B_I2225;BP1026B_I2226;BP1026B_I2227;BP1026B_I2229	UP
27	2514959	2529685	+	BP1026B_I2270;BP1026B_I2272;BP1026B_I2276;BP1026B_I2277;BP1026B_I2278;BP1026B_I2279;BP1026B_I2280;BP1026B_I2281	UP
28	2686538	2720977	-	BP1026B_I2423;BP1026B_I2424;BP1026B_I2425;BP1026B_I2426;BP1026B_I2429;BP1026B_I2432;BP1026B_I2435;BP1026B_I2437;BP1026B_I2438;BP1026B_I2444;BP1026B_I2445;BP1026B_I2446;BP1026B_I2447;BP1026B_I2448;BP1026B_I2449;BP1026B_I2450	UP
29	2998586	3038900	-	BP1026B_I2704;BP1026B_I2706;BP1026B_I2707;BP1026B_I2708;BP1026B_I2713;BP1026B_I2715;BP1026B_I2718;BP1026B_I2719;BP1026B_I2720;BP1026B_I2721;BP1026B_I2722;BP1026B_I2723;BP1026B_I2724;BP1026B_I2725;BP1026B_I2730;BP1026B_I2731;BP1026B_I2733;BP1026B_I2735;BP1026B_I2736;BP1026B_I2737	UP
30	3003904	3040258	+	BP1026B_I2709;BP1026B_I2710;BP1026B_I2711;BP1026B_I2712;BP1026B_I2716;BP1026B_I2726;BP1026B_I2727;BP1026B_I2728;BP1026B_I2729;BP1026B_I2732;BP1026B_I2734;BP1026B_I2738	UP
31	3281284	3289470	+	BP1026B_I2939;BP1026B_I2940;BP1026B_I2941	DOWN
32	3331732	3350602	+	BP1026B_I2982;BP1026B_I2984;BP1026B_I2985;BP1026B_I2986;BP1026B_I2987;BP1026B_I2988;BP1026B_I2989;BP1026B_I2993;BP1026B_I2994;BP1026B_I2995;BP1026B_I2997;BP1026B_I2998;BP1026B_I2999;BP1026B_I3001	UP
33	3366709	3386721	+	BP1026B_I3012;BP1026B_I3013;BP1026B_I3014;BP1026B_I3015;BP1026B_I3016;BP1026B_I3017;BP1026B_I3018;BP1026B_I3019;BP1026B_I3020;BP1026B_I3021;BP1026B_I3022;BP1026B_I3024;BP1026B_I3023;BP1026B_I3025;BP1026B_I3026;BP1026B_I3027;BP1026B_I3028;BP1026B_I3029;BP1026B_I3030;BP1026B_I3031	DOWN
34	3450617	3477500	+	BP1026B_I3090;BP1026B_I3095;BP1026B_I3106;BP1026B_I3107;BP1026B_I3108;BP1026B_I3109;BP1026B_I3110;BP1026B_I3117	UP
35	3516444	3543482	+	BP1026B_I3149;BP1026B_I3157;BP1026B_I3158;BP1026B_I3159;BP1026B_I3160;BP1026B_I3161;BP1026B_I3162;BP1026B_I3166;BP1026B_I3167;BP1026B_I3168;BP1026B_I3169;BP1026B_I3171;BP1026B_I3172	DOWN
36	3620485	3634475	+	BP1026B_I3243;BP1026B_I3244;BP1026B_I3245;BP1026B_I3246;BP1026B_I3251;BP1026B_I3253;BP1026B_I3254;BP1026B_I3255;BP1026B_I3256;BP1026B_I3257	UP
37	3766881	3792553	+	BP1026B_I3390;BP1026B_I3403;BP1026B_I3405;BP1026B_I3406;BP1026B_I3411;BP1026B_I3413	UP
38	3829144	3884401	-	BP1026B_I3458;BP1026B_I3460;BP1026B_I3470;BP1026B_I3471;BP1026B_I3480;BP1026B_I3482;BP1026B_I3484;BP1026B_I3489;BP1026B_I3494;BP1026B_I3496;BP1026B_I3497;BP1026B_I3499;BP1026B_I3501;BP1026B_I3504;BP1026B_I3505;BP1026B_I3506;BP1026B_I3507;BP1026B_I3509;BP1026B_I3513;BP1026B_I3515;BP1026B_I3517	UP
39	3840193	3847401	+	BP1026B_I3472;BP1026B_I3473;BP1026B_I3474;BP1026B_I3475;BP1026B_I3476;BP1026B_I3477;BP1026B_I3478;BP1026B_I3479;BP1026B_I3481	DOWN
40	4068843	4071726	-	BP1026B_I3702;BP1026B_I3703;BP1026B_I3704	DOWN
