# Supplementary material for: TetR-like regulator BP1026B_II1561 controls aromatic amino acid biosynthesis and intracellular pathogenesis in Burkholderia pseudomallei
Source: Front Microbiol. 2024 Aug 15;15:1441330. doi: 10.3389/fmicb.2024.1441330 (PMC11358695; doi:10.3389/fmicb.2024.1441330)
Supplement: SUPPLEMENTARY FIGURE S2 — WoPPER analysis reveals 50 gene clusters regulated by BP1026B_II1561 on Bp 1026b chromosome II. (A) Circular map of Bp 1026b chromosome II showing up-regulated (yellow) and down-regulated (blue) gene clusters of BP1026B_II1561. The outer circle is the plus strand (dark grey) and the inner circle is the negative strand (light grey). (B) Linear map of BP1026B_II1561 regulated gene clusters showing the mean log2FC of each cluster versus its position on chromosome II. The orange line represents the plus strand and the blue line represents the negative strand. The size of each gene cluster circle represents the number of genes within that given cluster. (C) Table summarizing the gene clusters regulated by BP1026B_II1561 on chromosome II. [file Image_2.pdf]

a b

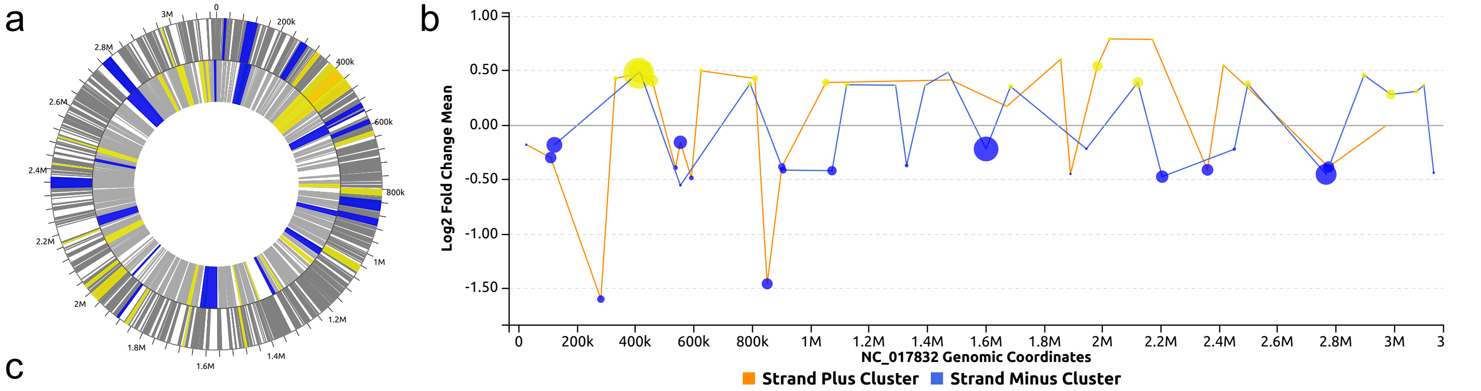

c

Cluster	Start Coordinates	End Coordinates	Strand	Gene ID	Expression
1	24146	29691	+	BP1026B_II0026;BP1026B_II0027;BP1026B_II0028;BP1026B_II0029;BP1026B_II0031	DOWN
2	94352	125583	+	BP1026B_II0084;BP1026B_II0085;BP1026B_II0088;BP1026B_II0091;BP1026B_II0093;BP1026B_II0098;BP1026B_II0099;BP1026B_II0100;BP1026B_II0102;BP1026B_II0103;BP1026B_II0104;BP1026B_II0105;BP1026B_II0106;BP1026B_II0107;BP1026B_II0108;BP1026B_II0109;BP1026B_II0110;BP1026B_II0111	DOWN
3	99792	142814	-	BP1026B_II0090;BP1026B_II0092;BP1026B_II0094;BP1026B_II0096;BP1026B_II0097;BP1026B_II0101;BP1026B_II0116;BP1026B_II0117;BP1026B_II0118;BP1026B_II0119;BP1026B_II0120;BP1026B_II0121;BP1026B_II0122;BP1026B_II0124	DOWN
4	269328	289984	+	BP1026B_II0224;BP1026B_II0225;BP1026B_II0229;BP1026B_II0230;BP1026B_II0231;BP1026B_II0232;BP1026B_II0234;BP1026B_II0235;BP1026B_II0236;BP1026B_II0237;BP1026B_II0238	DOWN
5	323939	337210	+	BP1026B_II0265;BP1026B_II0266;BP1026B_II0274;BP1026B_II0275;BP1026B_II0276	UP
6	374520	458474	-	BP1026B_II0307;BP1026B_II0308;BP1026B_II0310;BP1026B_II0311;BP1026B_II0312;BP1026B_II0314;BP1026B_II0318;BP1026B_II0319;BP1026B_II0324;BP1026B_II0325;BP1026B_II0327;BP1026B_II0328;BP1026B_II0329;BP1026B_II0330;BP1026B_II0331;BP1026B_II0332;BP1026B_II0333;BP1026B_II0334;BP1026B_II0335;BP1026B_II0336;BP1026B_II0337;BP1026B_II0338;BP1026B_II0339;BP1026B_II0340;BP1026B_II0348;BP1026B_II0349;BP1026B_II0350;BP1026B_II0356;BP1026B_II0357;BP1026B_II0358	UP
7	377577	439709	+	BP1026B_II0309;BP1026B_II0313;BP1026B_II0315;BP1026B_II0316;BP1026B_II0317;BP1026B_II0320;BP1026B_II0321;BP1026B_II0322;BP1026B_II0323;BP1026B_II0326;BP1026B_II0341;BP1026B_II0342	UP
8	442157	474765	+	BP1026B_II0344;BP1026B_II0345;BP1026B_II0346;BP1026B_II0347;BP1026B_II0351;BP1026B_II0352;BP1026B_II0353;BP1026B_II0354;BP1026B_II0355;BP1026B_II0362;BP1026B_II0363;BP1026B_II0364;BP1026B_II0365;BP1026B_II0366;BP1026B_II0371;BP1026B_II0370;BP1026B_II0372	UP
9	529475	542392	+	BP1026B_II0429;BP1026B_II0430;BP1026B_II0431;BP1026B_II0433;BP1026B_II0435;BP1026B_II0436;BP1026B_II0439	DOWN
10	535197	571714	-	BP1026B_II0432;BP1026B_II0434;BP1026B_II0437;BP1026B_II0438;BP1026B_II0446;BP1026B_II0448;BP1026B_II0449;BP1026B_II0450;BP1026B_II0456;BP1026B_II0457;BP1026B_II0458;BP1026B_II0459	DOWN
11	551683	554448	+	BP1026B_II0452;BP1026B_II0453;BP1026B_II0454;BP1026B_II0455	DOWN
12	583763	596567	+	BP1026B_II0469;BP1026B_II0470;BP1026B_II0471;BP1026B_II0472;BP1026B_II0473;BP1026B_II0474;BP1026B_II0475;BP1026B_II0476;BP1026B_II0477;BP1026B_II0478;BP1026B_II0479;BP1026B_II0480	DOWN
13	620528	632482	+	BP1026B_II0502;BP1026B_II0504;BP1026B_II0505;BP1026B_II0506;BP1026B_II0507;BP1026B_II0508;BP1026B_II0509;BP1026B_II0510;BP1026B_II0511;BP1026B_II0512;BP1026B_II0513;BP1026B_II0514	UP
14	783879	797131	-	BP1026B_II0631;BP1026B_II0632;BP1026B_II0639;BP1026B_II0641;BP1026B_II0642;BP1026B_II0643	UP
15	800331	818579	+	BP1026B_II0645;BP1026B_II0646;BP1026B_II0647;BP1026B_II0648;BP1026B_II0649;BP1026B_II0650;BP1026B_II0651;BP1026B_II0652	UP
16	834697	864763	+	BP1026B_II0670;BP1026B_II0681;BP1026B_II0683;BP1026B_II0682;BP1026B_II0684;BP1026B_II0685;BP1026B_II0686;BP1026B_II0690;BP1026B_II0691;BP1026B_II0694	DOWN
17	891031	911114	+	BP1026B_II0720;BP1026B_II0721;BP1026B_II0722;BP1026B_II0723;BP1026B_II0725;BP1026B_II0726;BP1026B_II0729;BP1026B_II0730;BP1026B_II0731;BP1026B_II0732;BP1026B_II0733;BP1026B_II0734;BP1026B_II0736;BP1026B_II0738;BP1026B_II0740;BP1026B_II0741	DOWN
18	894813	913775	-	BP1026B_II0724;BP1026B_II0727;BP1026B_II0728;BP1026B_II0735;BP1026B_II0737;BP1026B_II0739;BP1026B_II0743	DOWN
19	1041069	1061923	+	BP1026B_II0848;BP1026B_II0849;BP1026B_II0852;BP1026B_II0853;BP1026B_II0851;BP1026B_II0855;BP1026B_II0857;BP1026B_II0859;BP1026B_II0858;BP1026B_II0860;BP1026B_II0861;BP1026B_II0862;BP1026B_II0864;BP1026B_II0865;BP1026B_II0866	UP
20	1058329	1083898	-	BP1026B_II0863;BP1026B_II0875;BP1026B_II0876;BP1026B_II0877;BP1026B_II0878;BP1026B_II0879;BP1026B_II0880;BP1026B_II0884	DOWN
21	1117827	1132188	-	BP1026B_II0907;BP1026B_II0911;BP1026B_II0912;BP1026B_II0913;BP1026B_II0916;BP1026B_II0918;BP1026B_II0920;BP1026B_II0921;BP1026B_II0922	UP
22	1288775	1294261	-	BP1026B_II1062;BP1026B_II1065;BP1026B_II1066	UP
23	1321796	1331951	-	BP1026B_II1090;BP1026B_II1091;BP1026B_II1092;BP1026B_II1093;BP1026B_II1094;BP1026B_II1095;BP1026B_II1096;BP1026B_II1097;BP1026B_II1098;BP1026B_II1099;BP1026B_II1101	DOWN
24	1391271	1393392	-	BP1026B_II1109;BP1026B_II1110;BP1026B_II1111	UP
25	1467188	1470483	-	BP1026B_II1178;BP1026B_II1180;BP1026B_II1181	UP
26	1476021	1481920	+	BP1026B_II1184;BP1026B_II1185;BP1026B_II1186;BP1026B_II1187;BP1026B_II1188;BP1026B_II1189	UP
27	1567554	1634866	-	BP1026B_II1258;BP1026B_II1259;BP1026B_II1260;BP1026B_II1261;BP1026B_II1262;BP1026B_II1263;BP1026B_II1264;BP1026B_II1265;BP1026B_II1266;BP1026B_II1267;BP1026B_II1268;BP1026B_II1269;BP1026B_II1273;BP1026B_II1278;BP1026B_II1279;BP1026B_II1280;BP1026B_II1281;BP1026B_II1282;BP1026B_II1283;BP1026B_II1284;BP1026B_II1295;BP1026B_II1300;BP1026B_II1301;BP1026B_II1304	DOWN
28	1669880	1672481	+	BP1026B_II1329;BP1026B_II1332	UP
29	1676308	1689624	-	BP1026B_II1334;BP1026B_II1335;BP1026B_II1336;BP1026B_II1339;BP1026B_II1340;BP1026B_II1345	UP
30	1852747	1860940	+	BP1026B_II1464;BP1026B_II1469;BP1026B_II1470;BP1026B_II1472;BP1026B_II1471;BP1026B_II1473	UP
31	1885275	1891541	+	BP1026B_II1492;BP1026B_II1493;BP1026B_II1494;BP1026B_II1495;BP1026B_II1496;BP1026B_II1497;BP1026B_II1498	DOWN
32	1940433	1944617	-	BP1026B_II1534;BP1026B_II1535;BP1026B_II1536;BP1026B_II1537	DOWN
33	1967614	1996059	+	BP1026B_II1553;BP1026B_II1554;BP1026B_II1558;BP1026B_II1559;BP1026B_II1560;BP1026B_II1561;BP1026B_II1562;BP1026B_II1567;BP1026B_II1568;BP1026B_II1569;BP1026B_II1571;BP1026B_II1572;BP1026B_II1574;BP1026B_II1576;BP1026B_II1577;BP1026B_II1578	UP
34	2017814	2027829	+	BP1026B_II1595;BP1026B_II1596;BP1026B_II1597;BP1026B_II1598;BP1026B_II1599;BP1026B_II1600;BP1026B_II1602	UP
35	2104407	2133113	-	BP1026B_II1677;BP1026B_II1678;BP1026B_II1679;BP1026B_II1681;BP1026B_II1682;BP1026B_II1683;BP1026B_II1684;BP1026B_II1685;BP1026B_II1686;BP1026B_II1687;BP1026B_II1688;BP1026B_II1689;BP1026B_II1693;BP1026B_II1694;BP1026B_II1699	UP
36	2168126	2170715	+	BP1026B_II1733;BP1026B_II1734;BP1026B_II1735;BP1026B_II1736;BP1026B_II1738	UP
37	2186351	2220222	-	BP1026B_II1743;BP1026B_II1744;BP1026B_II1745;BP1026B_II1746	DOWN
38	2343359	2375470	+	BP1026B_II1852;BP1026B_II1857;BP1026B_II1858;BP1026B_II1859;BP1026B_II1864;BP1026B_II1870;BP1026B_II1878;BP1026B_II1879;BP1026B_II1880	DOWN
39	2414082	2416799	+	BP1026B_II1915;BP1026B_II1916;BP1026B_II1917;BP1026B_II1918	UP
40	2448471	2457240	-	BP1026B_II1950;BP1026B_II1951;BP1026B_II1953;BP1026B_II1956;BP1026B_II1957;BP1026B_II1958;BP1026B_II1959	DOWN
41	2487404	2505816	-	BP1026B_II1984;BP1026B_II1985;BP1026B_II1986;BP1026B_II1987;BP1026B_II1988;BP1026B_II1989;BP1026B_II1992;BP1026B_II1994;BP1026B_II1996;BP1026B_II2002	UP
42	2499725	2502336	+	BP1026B_II1995;BP1026B_II1997	UP
43	2735746	2792668	-	BP1026B_II2205;BP1026B_II2207;BP1026B_II2209;BP1026B_II2210;BP1026B_II2211;BP1026B_II2213;BP1026B_II2214;BP1026B_II2217;BP1026B_II2218;BP1026B_II2220;BP1026B_II2222;BP1026B_II2223;BP1026B_II2224;BP1026B_II2225;BP1026B_II2226;BP1026B_II2227;BP1026B_II2229;BP1026B_II2230;BP1026B_II2231;BP1026B_II2232;BP1026B_II2234;BP1026B_II2244;BP1026B_II2245;BP1026B_II2246;BP1026B_II2247;BP1026B_II2248;BP1026B_II2250	DOWN
44	2760314	2791272	+	BP1026B_II2216;BP1026B_II2221;BP1026B_II2228;BP1026B_II2233;BP1026B_II2235;BP1026B_II2236;BP1026B_II2238;BP1026B_II2239;BP1026B_II2249	DOWN
45	2889411	2903969	-	BP1026B_II2327;BP1026B_II2328;BP1026B_II2329;BP1026B_II2332;BP1026B_II2338;BP1026B_II2339	UP
46	2967523	2970419	+	BP1026B_II2399;BP1026B_II2400;BP1026B_II2402;BP1026B_II2403	UP
47	2974712	3001078	-	BP1026B_II2408;BP1026B_II2409;BP1026B_II2410;BP1026B_II2411;BP1026B_II2412;BP1026B_II2413;BP1026B_II2414;BP1026B_II2415;BP1026B_II2416;BP1026B_II2419;BP1026B_II2422;BP1026B_II2424;BP1026B_II2425;BP1026B_II2426;BP1026B_II2427;BP1026B_II2428;BP1026B_II2429;BP1026B_II2430;BP1026B_II2431;BP1026B_II2432	UP
48	3067872	3080761	-	BP1026B_II2482;BP1026B_II2483;BP1026B_II2484;BP1026B_II2485;BP1026B_II2487;BP1026B_II2488;BP1026B_II2489;BP1026B_II2490;BP1026B_II2491;BP1026B_II2492	UP
49	3094482	3103841	-	BP1026B_II2505;BP1026B_II2506;BP1026B_II2507;BP1026B_II2508	UP
50	3129738	3136611	-	BP1026B_II2528;BP1026B_II2531;BP1026B_II2535;BP1026B_II2536	DOWN
