# Supplementary figures and images for: TetR-like regulator BP1026B_II1561 controls aromatic amino acid biosynthesis and intracellular pathogenesis in Burkholderia pseudomallei
Source: Front Microbiol. 2024 Aug 15;15:1441330. doi: 10.3389/fmicb.2024.1441330 (PMC11358695; doi:10.3389/fmicb.2024.1441330)

a

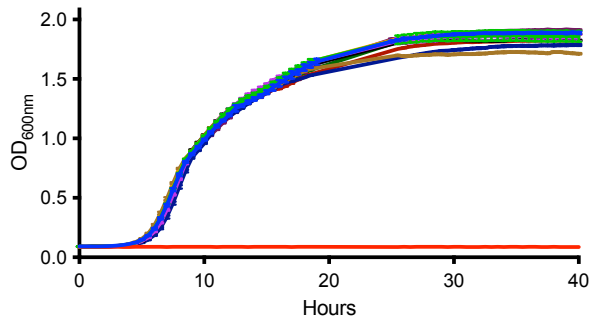

WT  
Media control  
*BP1026B\_I0063*  
*BP1026B\_I0071*  
*BP1026B\_I1775*  
*BP1026B\_I0229*  
*BP1026B\_I0230*  
*BP1026B\_I0232*  
*BP1026B\_I0681*  
*BP1026B\_I0684*  
*BP1026B\_I1562*

b

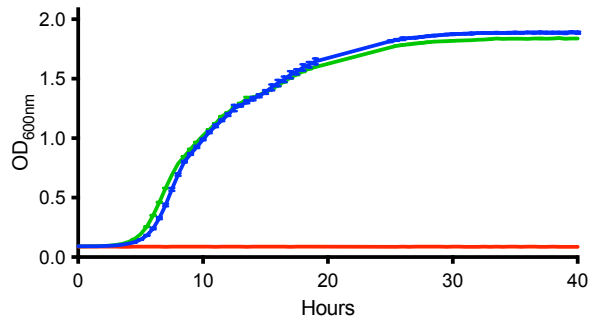

WT  
Media control  
*BP1026B\_I0782*

Supplement: SUPPLEMENTARY FIGURE S3 — Growth analysis of transposon mutants compared to wild type Bp 1026b. (A) Mutants indirectly controlled by BP1026B_II1561 that show defects during intracellular replication (Figures 4B,C) were tested for their ability to grow in LB. All mutants showed identical growth patterns to wild type Bp 1026b (WT) indicating that the defects during intracellular infection were not due to a defect in fitness. (B) A BP1026B_II0782 showed a significant defect during intracellular pathogenesis (Figures 6D,E) and shows no defect in fitness. [file Image_3.pdf]
